# Prevalence, Trends, and Socioeconomic Determinants of Coexisting Forms of Malnutrition Amongst Children under Five Years of Age in Pakistan

**DOI:** 10.3390/nu13124566

**Published:** 2021-12-20

**Authors:** Asif Khaliq, Darren Wraith, Yvette Miller, Smita Nambiar-Mann

**Affiliations:** 1 School of Public Health and Social Work, Queensland University of Technology, Brisbane 4059, Australia; d.wraith@qut.edu.au (D.W.); yvette.miller@qut.edu.au (Y.M.); 2 School of Exercise and Nutrition Sciences, Queensland University of Technology, Brisbane 4059, Australia; smita.nambiar@qut.edu.au

**Keywords:** malnutrition, coexisting form, prevalence, trends, determinants, socioeconomic, children, under five years, Pakistan

## Abstract

In Pakistan, malnutrition is a chronic issue. Concerns regarding coexisting forms of malnutrition (CFM) in an individual child are emerging, as children suffering from CFM have a 4 to 12-fold higher risk of death compared with healthy children. This study assessed the prevalence, trends, and socioeconomic determinants of various types of CFM using Pakistan Demographic and Health Survey (PDHS) datasets. Data from children aged 0–5 years old, with complete height and weight information, and valid anthropometry, from all regions of Pakistan (except residents of Azad Jammu Kashmir (AJK) and Federally Administered Tribal Areas (FATA), and non-de jure residents), were included. The prevalence of CFM was 30.6% in 2012–2013 and 21.5% in 2017–2018 PDHS. Both PDHSs reported a significantly higher prevalence of CFM in Sindh and Baluchistan compared with other regions of Pakistan. Improved socioeconomic status significantly reduced the odds of various types of CFM, except the coexistence of underweight with wasting. The high prevalence of CFM in Pakistan can be averted by multisectoral collaboration and by integrating nutrition-sensitive and nutrition-specific interventions.

## 1. Introduction

Malnutrition is a serious public health concern affecting at least half of children under five years of age in developing countries [1,2,3]. Stunting, wasting, underweight, overweight, and obesity are the most common types of malnutrition in children [4]. Children living in Asian and African regions of the world have the highest risk of various forms of malnutrition [5]. The prevalence of all forms of malnutrition in Pakistan, India, and Bangladesh has been over the acceptable threshold limits of 30% stunting, 15% wasting, and 10% underweight [5,6,7].

In the current era, there is global concern regarding coexisting forms of malnutrition (CFM) in the same child. For example, the simultaneous presence of stunting together with overweight/obesity represents a contrasting form of malnutrition, demanding interventions that can concurrently manage and control both undernutrition as well as overnutrition [8,9]. The 2018 and 2019 Global Nutrition Report identified two types of CFM in children: Coexistence of wasting with stunting, and of stunting with overweight/obesity [10], while the World Health Organization’s 2020 report described the simultaneous occurrence of stunting and/or wasting among underweight children [11]. Children suffering from more than one form of malnutrition had a 4 to 12-fold higher risk of death compared with healthy children [12].

Socioeconomic status represents the underlying basic cause of malnutrition and is composed of multiple variables such as type of sanitation facilities, source of drinking water, and housing infrastructure [13,14]. Poor socioeconomic status is directly related to illiteracy, unemployment, reduced purchasing power, and also to poor health and nutritional outcomes [15,16]. Studies conducted in China, Mexico, and Sub-Saharan African countries have shown a higher risk of coexistence of stunting with overweight/obesity in children of the low socioeconomic class than children of higher socioeconomic class. However, evidence regarding other forms of CFM (i.e., coexisting forms of undernutrition) is scarce [17,18,19]. Provision of various cultural and environmental support services, such as charity, donation, zakat (mandatory charity for poor Muslims), and basic health insurance, has the potential to avert issues related to poverty, and poor health and nutritional outcomes [15,20].

Malnutrition in Pakistan is a chronic issue, particularly in children under five years of age [21]. Economically, Pakistan is considered a Low-Middle Income Country (LMIC). In general, various behaviors and practices, such as dietary intake, care-seeking practices, and disease management, are related to socioeconomic status [22]. Different studies have also supported a strong relationship between socioeconomic status with pediatric nutritional status [23,24]. Still, the prevalence, trends, and determinants of various types of CFM are either scarce or not known. In this study, secondary data analysis of the Pakistan Demographic and Health Survey (PDHS) datasets were used to assess the prevalence, trends, and socioeconomic determinants of various types of CFM.

## 2. Materials and Methods

### 2.1. Datasets

In this study, secondary data analysis of the Pakistan Demographic and Health Survey (PDHS) datasets from 2012–2013 and 2017–2018 were conducted. The DHS is a nationally representative dataset, which assesses the demographic and health status of women of reproductive age and children below five years of age. Only the 2017–2018 survey collected data from all eight regions of Pakistan: Sindh, Punjab, Baluchistan, Khyber Pakhtunkhwa (KPK), Gilgit Baltistan (GB), Azad Jammu and Kashmir (AJK) and Federally Administered Tribal Areas (FATA) and Islamabad Capital Territory (ICT). The 2012–2013 PDHS excluded AJK and FATA regions due to geopolitical, military restriction and security reasons [25]. Thus, in this study, we excluded the AJK and FATA regions from the latter survey to make meaningful comparisons between the time points of interest. While the results from the latter survey are generally presented as nationally representative, it is difficult to determine the effect of the exclusion of AJK and FATA regions. Due to this, the results from this study may only be representing large regions of Pakistan.

### 2.2. Study Participants and Eligibility Criteria

This study analyzed the anthropometric data of children aged below five years of age after excluding non-de jure residents, and residents of AJK and FATA. Children with missing anthropometric variables (age, sex, height/length measurement, and weight measurement) or who were considered anthropometric outliers were excluded. The World Health Organisation described anthropometric outliers based on the biologically implausible z-score values: HAZ/LAZ ≥ 6.00 S.D (Standard Deviation). or ≤−6.00 S.D.; WHZ ≥ 5.00 S.D (Standard Deviation). or ≤−5.00 S.D.; and WAZ ≤ −6.00 or ≥5.00 S.D. These anthropometric outliers occur due to measurement or recording errors [26,27].

### 2.3. Sample Size and Sampling Strategy

**Figure 1:**
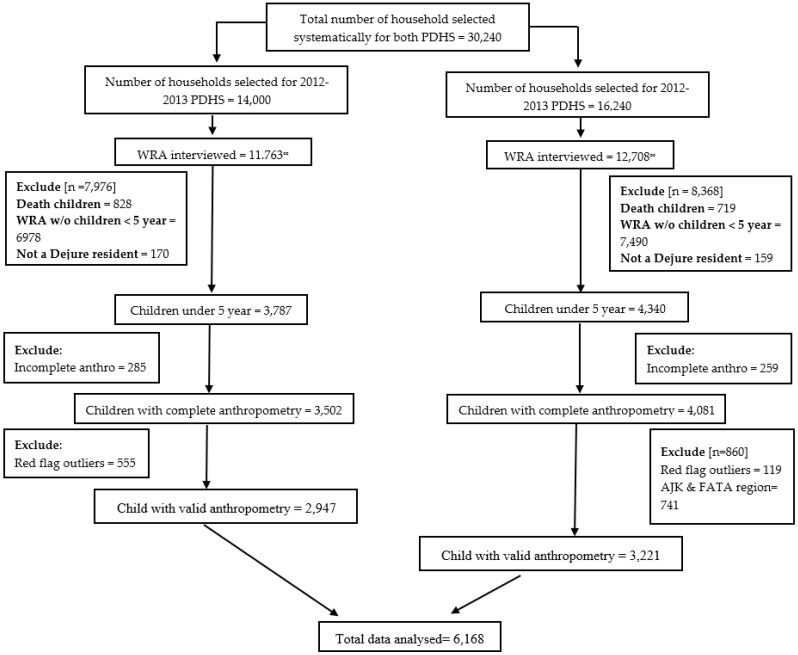
Child nutritional status assessment based on z-score and anthropometric indices. PDHS = Pakistan Demographic and Health Survey; WRA = Women of Reproductive Age; w/o = Without; Incomplete Anthro. = The dataset has missing information about the child age or sex, or weight, or length/height, or measurement method (standing/recumbent); ∞ = Number of WRA were less than the calculated sample size because of refusal and/or ineligibility and/or inaccessibility due to geopolitical/security issues; Red flag outliers = The z-scores calculated for HAZ/LAZ exceeds over ±6.00 S.D., WHZ exceeds over ±5.00 S.D., and of WAZ must exceed −6.00 and +5.00 S.D., respectively.

In each PDHS, the sample size was calculated based on the number of enumeration blocks (EB), which represents a cluster of around 200 to 250 households. Details regarding the EBs are outlined in the Pakistan Bureau of Statistics (PBS) and the Pakistan Population and Housing census. EBs were selected randomly according to their size. From each EB, a fixed number of 28 households were selected systematically. The systematic selection of 28 households from each EB held in the data processing office of National Institute of Population Studies (NIPS), and the survey team only approached pre-selected households for the interview. In the 2012–2013 PDHS, 500 EBs were selected, whereas in the 2017–2018 PDHS 580 EBs were considered, thus producing a total sample size of 14,000 and 16,240, respectively. From each pre-selected household, women of reproductive age (WRA) aged between 15 to 49.9 years were interviewed for data collection. The survey team excluded certain clusters due to military restrictions and security reasons. A total of 11,763 in 2012–2013 and 12,708 in 2017–2018 women were included after excluding those who were ineligible. Household’s where the survey team failed to find WRA despite several visits or/and where the WRA had either participated partly or refused to participate were considered ineligible [25,28,29]. Among the interviewed WRA, 3787 in 2012–2013 and 4340 in 2017–2018 had a child under five years of age. Removal of cases based on exclusions described above and in Figure 1 resulted in a total sample of 6168, of which 47.8% (2947) were from 2012–2013, and the remaining 52.2% (3221) were from the 2017–2018 PDHS. For determining the sample size adequacy of this study, post-hoc power was calculated for the total sample considering α = 0.05, the individual sample of each study, and the prevalence of pediatric malnutrition calculated from each survey. The post-hoc power calculated for this study was over 80% [30]. Details regarding the treatment of cases for inclusion are presented in Figure 1.

### 2.4. Data Collection Method and Data Collection Tool

In each PDHS, a team of qualified, experienced, and trained data collectors interviewed women of reproductive age from each selected household using four to six structured questionnaires. The Household Questionnaire, Man’s Questionnaire, Woman’s Questionnaire, and Community Questionnaire were used in both surveys, while two additional questionnaires, the Biomarker Questionnaire and Fieldworker Questionnaire, were used in the 2017–2018 survey. For this study, variables from the household questionnaire, women’s questionnaire, and the biomarker questionnaire were selected. The household questionnaire of each PDHS collected information about the basic living standards, including assessments of housing infrastructure, type of toilet facility, source of drinking water, and possession of common household assets, including land and property. Moreover, the 2012–2013 PDHS household’s questionnaire also collected data on child anthropometry. The 2017–2018 PDHS household’s questionnaire did not collect anthropometric data, rather a new questionnaire “biomarker questionnaire” collected anthropometric data in the 2017–2018 survey. The Woman’s questionnaire of each PDHS collected information related to a woman’s demography and health. The details regarding each survey questionnaire can be accessed from the Appendix-F of each PDHS report [25,29]. A team of data collectors including supervisors interviewed the targeted population using a set of these questionnaires. All data collectors and their supervisors involved in each PDHS received three to four weeks of training regarding interviewing technique, questionnaire probing, and collecting anthropometry. The accuracy and precision of anthropometric measurements performed by each data collector were assessed by comparing them to standardized anthropometric measurements. Further training was provided to those data collectors whose anthropometric measurements were not standardized. Field supervisors received additional training about data management, data quality, and field supervision [25,28,29].

### 2.5. Measurement of Outcome Variable

In each PDHS, a team of trained anthropometrists measured the weight and length/height of all children aged from 0 to 59 months (about five years), using calibrated and standardized weighing scales and stadiometer. In this study, information related to a child’s weight, height/length, age, and gender were imported to the World Health Organization’s Anthropometry Calculator (AnthroCalc 2006). The WHO (World Health Organization) AnthroCalc-2006 converted the raw height and weight values into the following z-scores: Weight-for-age (WAZ); length/height-for-age (LAZ/HAZ), and weight-for-length/height (WHZ). Based on the z-score values, children were classified as undernourished or overnourished. A cut-off value of −2.00 S.D or less was used for all the anthropometric indices for the diagnosis of undernutrition (stunting, or wasting, or underweight). Children having z-score for HAZ, WHZ, and/or WAZ below −2.00 S.D were considered stunted, wasted, and/or underweight, respectively. Children with z-score values over −1.99 S.D were classified as normal/healthy children, but for WHZ, a normal/healthy child must have a z-scores value ranging between −1.99 to +1.99 S.D. Any child having WHZ z-score value between +2.00 S.D. to +5.00 S.D. was considered overweight/obese. The nutritional status of each child was further analyzed through computational analysis to create nine categories of nutritional status: Normal, Wasting, Stunting, Underweight, Overweight/Obesity and the following CFM: Underweight with stunting, underweight with wasting, underweight with both wasting and stunting, and stunting with overweight/obesity (nutritional paradox) (Further detail—S1).

### 2.6. Conceptual Framework

**Figure 2:**
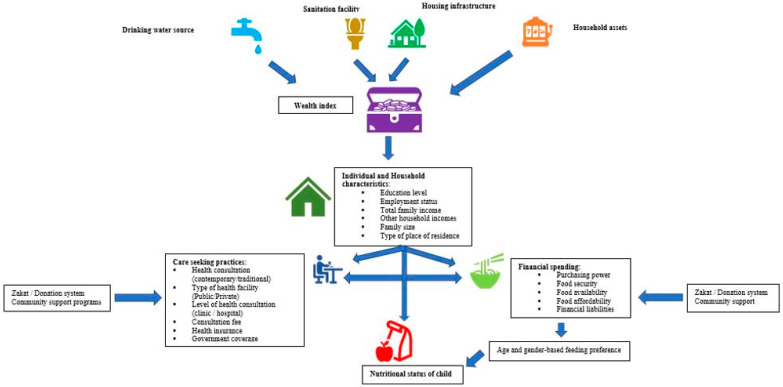
Conceptual framework defining the relationship of pediatric malnutrition with socioeconomic status and related factors.

CFM is a novel concept. Like standalone forms of malnutrition, CFM has multiple determinants, extending from individual biology to maternal, household, and nationwide factors. The conceptual framework designed by UNICEF (United Nation International Children Emergency Funds) for malnutrition has three tiers: Immediate, underlying, and basic. Poor socioeconomic status, also called poverty, is related to illiteracy and unemployment, which in turn affects food purchasing power and affordability of healthcare services [15,16]. Poverty is also linked with poor health and nutritional outcomes particularly in young children aged below five years of age [15,20]. Figure 2 illustrates the relationship between pediatric malnutrition and socioeconomic and other related factors.

### 2.7. Study Covariates

The PDHS datasets contained information related to the individual, households, and care-seeking behaviors, but were devoid of information related to total family income. Initially, we considered all individual, household, and care-seeking factors as potential covariates for assessing the relationship of CFM with socioeconomic status. The variables related to care-seeking practices were excluded following preliminary analysis due to the presence of missing values. Thus, based on the above conceptual framework, child age, child gender, parental education, employment status, family size, and place of residence were identified as potential covariates from each PDHS dataset.

Age and sex represent the basic biology of an individual. To identify the differential risk of CFM in children under five years of age, we divided child age into five categories: 0 to 11 months, 12 to 23 months, 24 to 35 months, 36 to 47 months, and 48 to 59 months. Sex was dichotomized as male or female. Parental education and employment status of each parent were considered, resulting in four variables: Maternal education, paternal education, maternal employment status, and paternal employment status. In each PDHS, maternal and paternal education is recorded as one of four categories: None, primary, secondary, and higher, but in this study, the last two educational categories (secondary and higher) were merged, to distinguish between education at the primary level or beyond the primary level [31]. Maternal and paternal employment status was recorded dichotomously as: Yes or No. Family size was recorded continuously and dichotomized as 1 to 7 family members or 8 or more family members. The place of residence was dichotomized as Urban or Rural.

### 2.8. Measurement of Predictor Variable

Socioeconomic status was the primary predictor variable used in our analysis. This composite variable consisted of accessibility to safe drinking water, accessibility to sanitary latrines, floor material, and ownership of household items, including electricity, radio, television, refrigerator, bicycle, motorcycle, car, truck, mobile phone, washing machine, and water pump. Principal Component Analysis (PCA) was used to derive socioeconomic status in each PDHS. The socioeconomic status for each individual was labeled as poorest, poorer, middle, richer, and richest based on their computed score [32].

### 2.9. Statistical Analysis and Inference

Descriptive analysis was performed to assess the distribution of each categorical and numerical variable. Inferentially, chi-square and independent-sample t-test were performed for assessing the difference in the distribution of predictor and covariate variables across two survey periods. Differences in the distribution of these variables between survey periods were considered significant at *p* ≤ 0.05.

For assessing the geographic distribution and trends of malnutrition and its various types, prevalence estimates for each type of malnutrition were calculated at the national and regional levels. The 2012–2013 PDHS did not collect data from the AJK and FATA regions, therefore data from these regions were left out of the main analysis to make both datasets comparable. However, prevalence estimates for these regions have been calculated and presented in Supplementary File S4. The calculated prevalence estimates for each region were then converted into percentages. The prevalence estimates for each region were compared using 95% confidence interval limits, i.e., the regional prevalence estimates of a region were considered significantly different if their confidence interval limit did not overlap with the lower and upper 95% confidence interval limit value of the other regions’ prevalence estimates. Differences between times of assessment in malnutrition and its various types were assessed similarly.

The outcome of this study was the presence or absence of four types of CFM: Coexistence of underweight with stunting, the coexistence of underweight with wasting, the coexistence of underweight with wasting and stunting, and coexistence of stunting with overweight/obesity (nutritional paradox). The coexistence of either stunting or wasting or both with underweight is collectively referred to as coexisting forms of undernutrition (CFU). For each type of CFU, we selected underweight children as the referent category, because different studies supported the coexistence of wasting, or stunting, or both in underweight children [11,33]. For the nutritional paradox, stunting was chosen as the referent category, and many studies reported the prevalence of pediatric overweight/obesity among stunted children [34].

Due to the nominal and categorical nature of the outcome variables, we performed unordered logistic regression for examining the unadjusted odds ratio (OR) for each factor associated with each type of CFM. Low Variance Inflation Factor (VIF) of less than 2.0 and tolerance of less than 1.0 indicated an absence of multicollinearity in all models. The adjusted OR for each outcome was then calculated after controlling for covariates using a series of multivariable logistic regression models. Socioeconomic status was retained as the predictor with backward elimination of factors without a significant association with the outcomes. Variables without a significant association (*p*-value > 0.05) were removed sequentially, and only those variables having *p*-value ≤ 0.05 were retained in the final model. The OR and 95% confidence interval (CI) for each variable were calculated to measure the association of different covariates with various forms of CFM. For assessing the determinants of each form of CFM, we merged the datasets. Moreover, sensitivity analysis was performed for each study outcome with each study dataset (Supplementary File S3). To assess variations in associations of different covariates with each outcome over time, we also measured the interaction of covariates with the year of survey for each outcome variable. In our study, there were several outcomes measured. Initially, we tested the interaction of socioeconomic status, and all significant variables with the survey year but it was showing evidence of overfitting, and interaction factors were not significant. For this reason, we examined the interaction of each covariate with survey year for each outcome separately rather than from the adjusted model.

### 2.10. Ethics

The data used in this study was obtained from the DHS repository and the protocol of this study was approved by the University Health Research Ethics Committee (UHREC), Queensland University of Technology (QUT), Brisbane, Australia (Approval number 2000000177).

## 3. Results

### 3.1. Characteristics of Study Population—PDHS 2012–2013 and PDHS 2017–2018 Datasets

This study assessed the nutritional status of 6168 children under the age of five years using PDHS of 2012–2013 (*n* = 2947) and 2017–2018 (*n* = 3221).

Around half of the participants (41.5% in 2012–13 and 43.6% in 2017–2018) were from the poorer/poorest socioeconomic classes. Similarly, the 2017–2018 survey reported lower participation from the richer/richest socioeconomic class, compared to the 2012–2013 survey (40.8% in 2012–2013 to 37.7% in 2017–2018).

We found a significant difference in maternal education (*p* < 0.001) and maternal employment (*p* < 0.001) over the five to six-year period. In both surveys, more than half of the mothers had no education. There was a decrease in maternal illiteracy alongside an increase in maternal education at the beyond primary (secondary/higher) level between surveys. Maternal employment was 20.8% in the 2012–2013 survey, which decreased significantly to 12.5% in the 2017–2018 survey (<0.001). More than 50% of fathers in both surveys had received secondary/higher education, and more than 95% were employed.

More than half of households have at least eight members in their family. Around half of the participants had fully constructed housing infrastructure, and around 90% had access to safe drinking water in both PDHSs. The 2017–2018 survey showed a significant improvement in the access to toilet and sanitation facilities to 81.7% from 74.9% in 2012–2013 (<0.001). Similarly, the 2017–2018 survey reported a significant increase in the influx of urban residence, compared with the 2012–2013 PDHS (*p* < 0.001). More than half of the participants were from rural areas (Table 1).

### 3.2. National Prevalence and Trends of CFM

The national prevalence of child malnutrition was significantly lower in 2017–2018 (43.3%; 95% CI: 41.5% to 45%) compared to 2012–2013 (54.4%; 95% CI: 52.6% to 56.2%). The national prevalence of CFM in 2012–2013 was 30.6% (27.1% to 34.5%), while in 2017–2018 it decreased to 21.5% (20.1 to 23%). The coexistence of underweight with stunting was evident in more than 10% of children. The prevalence of other types of CFM was less than 5%. The 2017–2018 PDHS showed a significant reduction in the national prevalence of various forms of CFM compared to 2012–2013 PDHS (Table 2).

### 3.3. Regional Distribution of Various Types of Malnutrition

Both surveys reported a significantly higher prevalence of malnutrition in the Baluchistan and Sindh province, compared to national malnutrition estimates. Across two survey periods, Punjab, Sindh, Baluchistan, and Gilgit Baltistan province reported a significant decline in the prevalence of pediatric malnutrition in 2017–2018, compared to the former survey of 2012–2013. Despite a significant decline in malnutrition, still over half of the children of Baluchistan, and Sindh were found to be malnourished in the 2017–2018 survey (see Table 2).

Compared to the 2012–2013 PDHS, all the provinces and regions of Pakistan reported a significant decline in the prevalence of CFM in 2017–2018, except GB and KPK. Compared to national nutritional estimates, Sindh and Baluchistan showed a significantly higher prevalence of CFM in both surveys. Within Sindh and Baluchistan provinces, CFM is present in more than a third of children below five years of age.

The 2017–2018 survey showed a significant decline in the prevalence of coexisting forms of undernutrition in Sindh, Punjab, and KPK provinces, compared to the 2012–2013 survey. However, the Baluchistan and GB province showed a significant decline in the prevalence of nutritional paradox in the 2017–2018 survey compared to 2012–2013.

The geographical vulnerability to various forms of CFM varied over time in different provinces and regions of Pakistan. The 2012–2013 survey reported the highest prevalence of coexisting forms of undernutrition in Sindh, while in 2017–2018 reported the highest prevalence of coexisting forms of undernutrition in Baluchistan. Regarding nutritional paradox, the highest prevalence was reported from Baluchistan in 2012–2013, and GB province in 2017–2018 (Table 2). The graphical presentation of various types of Malnutrition, including CFM can be accessed from the Supplementary File S2.

### 3.4. Determinants of Coexistence of Underweight with Wasting

Compared with children of the poorest socioeconomic class, we found a 64% (13% to 85%) lower odds of coexistence of underweight with wasting in children from the richest socioeconomic class, after adjustment for other significant covariates. Among different child, parental, household, and periodic determinants, maternal working status was significantly associated with the coexistence of underweight with wasting. We found 53% (5% to 77%) lower odds of coexistence of underweight with wasting in children of working mothers, compared to non-working mothers. Across two survey periods, we did not find a significant interaction of any covariate with the coexistence of underweight with wasting (Table 3).

### 3.5. Determinants of Coexistence of Underweight with Stunting

Socioeconomic status was significantly associated with the coexistence of underweight with stunting, after adjusting for other determinants. We observed an 82% (59% to 92%) lower odds of coexistence of underweight with stunting from the children of the richest class, compared with children from the poorest socioeconomic strata.

The multivariable analysis showed over five-fold higher odds of coexistence of underweight with stunting in children aged over 11.9 months, compared with children aged between 0 to 11.9 months.

Across the two survey periods, the 2017–2018 survey (compared to the 2012–2013 survey) showed a significantly higher (8.82, 95% CI: 4.01 to 19.43) prevalence of coexistence of underweight with stunting in children aged 36–47 months, compared with children aged below 12 months. (Table 3).

### 3.6. Determinants of Coexistence of Underweight with Both Stunting and Wasting

Multivariable analysis revealed a significant association of socioeconomic status with the coexistence of underweight with wasting and stunting both (Table 4). Among various child, maternal, and household factors, we found a higher odds for the coexistence of underweight with wasting and stunting both in children aged over 11.9 months.

Across the two survey periods, we did not observe a significant interaction of any covariate with the coexistence of underweight with both wasting and stunting (Table 3).

### 3.7. Determinants of Coexistence of Stunting with Overweight/Obesity

Compared to children from poorer socioeconomic class, we found 50% (21% to 68%), 52% (22% to 71%), and 61% (34% to 78%) lower odds of coexistence of stunting with overweight/obesity in children of the poorer, middle, and richer socioeconomic classes, respectively. Children in the 2017–2018 survey had a 78% (68% to 85%) lower odds of coexistence of stunting with overweight/obesity, compared with those in the 2012–2013 survey. Children aged over 1 year, and with maternal employment showed a significantly lower odds of coexistence of stunting with overweight/obesity, compared to infants, and children with non-working mothers. However, we found higher odds of coexistence of stunting with overweight/obesity among children residing in urban Pakistan (Table 4).

Across two survey periods, the 2017–2018 survey compared to the former survey of 2012–2013 showed around 80% (22% to 95%) lower odds of coexistence of stunting with overweight/obesity in children aged between 36–47 months (about 4 years). The 2017–2018 survey (compared with 2012–2013) survey showed more than five-fold higher the odds of coexistence of stunting with overweight/obesity in children from upper socioeconomic class, compared to children of poorest socioeconomic class. Similarly, children of educated mothers (compared to uneducated mothers) showed over two-fold higher odds of coexistence of overweight/obesity in the 2017–2018 survey, compared to the 2012–2013 survey (Table 3).

## 4. Discussion

This study assessed the prevalence, trends, and socioeconomic determinants of various types of CFM in children under the age of five years across Pakistan using largely representative datasets from the PDHSs in 2012–2013 and 2017–2018. Historically, all previous surveys conducted in Pakistan and other parts of the world have assessed the prevalence, trends, and determinants of various standalone forms of nutrition and one type of CFM-stunting with overweight and obesity [25,29,35,36,37]. There are only a few studies conducted in Bangladesh [38], and Brazil [39], where determinants for various types of coexisting forms of undernutrition have been investigated [35,36,37]. Thus, the determinants of other types of coexisting forms of undernutrition (coexistence of underweight with wasting, coexistence of underweight with stunting, and coexistence of underweight with wasting and stunting) have not been investigated. In Pakistan, nearly half of the children under the age of five are malnourished. Among malnourished children, CFM affects nearly a half to two-thirds of children. CFM in children is highly attributed to various forms of undernutrition, which is responsible for around half of child mortality [40,41,42]. Moreover, children suffering from CFM have more than a ten-fold higher risk of infections and deaths than those with standalone forms of malnutrition [12].

We found that improved socioeconomic status was associated with significant reductions in the odds of various forms of CFM. While there is convincing evidence for associations between socioeconomic status and standalone forms of malnutrition [43,44,45], the relationship of socioeconomic status with CFM is under-researched. Most of the developing countries, including Pakistan, are confronting issues related to economic crises, such as poverty, social insecurities, inflation, unemployment, and food insecurity [45,46]. In Pakistan, where more than two-thirds of the population are food insecure, this food insecurity together with poverty is a leading cause for the escalated prevalence of pediatric malnutrition [45]. In this regard, special emphasis needs to be given to food insecurity, purchasing power, inflation, and socioeconomic status of country residents while devising policies and interventions for the prevention and control of malnutrition, including CFM.

This study reported a significant improvement in the nutritional profile of the children of Pakistan across two surveys period from 2012–2013 to 2017–2018. The exact reason for the changing trend of pediatric nutrition status during these surveys is difficult to determine. This is partly due to non-uniform and fragmented data collection history relating to health and nutrition indicators. We postulate several reasons for the changing trend during this period. Factors including health, education, housing infrastructure, agriculture production, labor force, and industrialization are prime indicators for assessing the socio-economic development of a nation [47]. The observed differences in the two survey periods can be partially explained due to changes in these socioeconomic characteristics of the study population. The 2017–2018 PDHS reported a significant improvement in socioeconomic indicators, including improvements in maternal education, clean toilet facilities, constructed housing infrastructure, and urban influx compared to the former PDHS of 2012–2013. A regional-based study conducted in Pakistan also showed a strong association between socio-economic development and better health and nutritional outcomes [23].

Despite a significant socioeconomic development reported from 2017–2018 survey, we observed a significant decline in maternal employment in 2017–2018 from the survey in 2012–2013. Similarly, the Pakistan Labor Force Survey (PLFS) reported a marked improvement in women’s education and a significant decline in women’s employment between 1984 to 2017 [48]. The reasons for this decrease in maternal employment are not clear. We can only speculate that Pakistan is still largely a patriarchal society where women are overrepresented in homecare [49]. The proportion of maternal education compared to paternal education is also low in both surveys, and education is a key for finding regular employment. Similarly, another study identified large family size, joint family structure, and a high number of children hinders women’s employment in Pakistan [50].

Other reasons for changes in the nutritional outcome and socioeconomic outcomes across the two survey periods are difficult to determine as a number of counteracting factors are involved. In 2010, Pakistan experienced massive flooding across all of its major provinces, and this historic flooding affected around 20 million (10%) residents of Pakistan [51,52]. The 2010 flood had long-lasting effects, which lasted from a few weeks to around six months and caused significant socioeconomic devastation, such as loss of basic life necessities, mass destruction of life stocks and agriculture, unemployment, poverty, and food insecurity [51,53,54,55]. Casare, et al., (2015) speculated that a high prevalence of malnutrition in women and children of Pakistan was a consequence of natural disasters, such as the 2010 flood [56]. In contrast, for the last ten years (2010 to 2019), Pakistan has made significant improvements to tackle the escalated prevalence of maternal and child malnutrition, and this includes increased allocation of GDP on health (from 2.5 to 3.2), food regulatory authority development, intersectoral nutritional strategies, investment in food fortification, the establishment of public-private partnership (PPP), and increased collaboration with World Bank, United Nations, and other regulatory bodies, such as Department of International Development, United Kingdom [57,58,59,60,61,62]. However, other health and nutrition surveillance have depicted either stagnancy or escalation in the prevalence of various forms of malnutrition. The National Nutrition Survey (NNS) of 2018 showed an exceptionally high prevalence of all forms of malnutrition compared to the former NNS of 2011, and of the 2017–2018 PDHS [25,63]. Moreover, the nutritional estimates of current Multiple Indicator Cluster Surveys (MICSs) reported either stagnancy or escalation in the prevalence of all forms of malnutrition, compared with the former MICS [64,65,66,67]. Thus, in Pakistan, it is uncertain as to whether pediatric nutritional status is either improving or deteriorating over time. For tackling the escalated prevalence of malnutrition, including CFM, the Ministry of planning and development, Pakistan has set annual targets for malnutrition reduction. An integration of nutritional sensitive programs (improving existing toilet facilities, sanitation, hygiene, and water treatment), with nutritional specific programs (exclusive breastfeeding, complementary feeding, maternal and child micronutrient supplementation, and cash transfer) can help to reduce the prevalence of different forms of malnutrition [45,62,68].

This study identified a persistently higher prevalence of malnutrition, including CFM in the Sindh and Baluchistan provinces compared to the rest of the country. Within Sindh and Baluchistan province, one out of every three children experienced CFM, while in other regions of Pakistan less than a quarter of children experienced CFM. The current estimates from National Nutritional Surveys (NNSs) [63] and other studies [69] also reported a higher prevalence of malnutrition in Sindh and Baluchistan province, with around half of the children suffering from malnutrition. Owing to recurrent natural catastrophizes, Sindh province has been declared as a region of nutritional emergency. After 2010 nationwide flooding, the Sindh province experienced another episode of massive flooding in 2011 [70]. Moreover, in 2013, a severe form of drought-hit the Tharparker region of Sindh, causing deaths of over 1500 children in a year [55,71,72]. Similarly, a lack of sanitation and hygiene, unclean drinking water supply, scarcity of livestock and agriculture products serve as a prime reason for the escalated prevalence of malnutrition in Baluchistan province [69,73]. For tackling the uncontrolled cases of malnutrition, the provincial government of Sindh and Baluchistan established a nutrition wing and initiated a rural support program which aims to reduce malnutrition by 75% by 2026 [45,73].

The issues related to various forms of CFM emerge in children after the infancy period (>11.9 months). We found a significantly higher odds of coexisting forms of undernutrition in children after their first birthday. Conversely, an increase in the age of a child serves to protect them from the coexistence of stunting with overweight/obesity. A study conducted in Indonesia also found a decreased risk of coexistence of stunting with overweight/obesity in older children, compared with younger children [74]. Thus, it can be concluded that children after the age of one year are prone to rapid increases in the risk of various forms of undernutrition, including coexisting forms of undernutrition. This rapid increase in the risk of various forms of undernutrition, including coexisting forms of undernutrition raises concern regarding the high risk of morbidity and mortality [44,45,75]. The first thousand days of life from conception to the second birthday provides a critical opportunity for the prevention and control of various types of health and nutritional disorders, as after this period children may experience irreversible growth retardation [75,76]. Hence, an integrated approach for the prevention and control of the various types of CFM needs to be devised specifically during the first thousand days of life [45,61,76].

This study found that maternal employment is protective against various forms of malnutrition. Previous studies conducted have shown a protective effect of maternal education and maternal employment, and a negative effect of large family sizes [23,75,77]. An improvement in maternal education boosts maternal knowledge regarding feeding practices, disease management, and care-seeking behaviour. Maternal education, when coupled with maternal employment supports maternal empowerment, which in turn improves socioeconomic status [77]. Improvement in socioeconomic status is fundamental for improving the nutritional status of children. Priority should be given to improving socioeconomic status by improving maternal and paternal education, creating employment opportunities, and developing conditions for healthier living, such as improved accessibility to essential foods, access to safe drinking water, hygienic sanitation facilities, and constructed housing infrastructure. Thus, a holistic approach can alleviate various forms of CFM through joint efforts and multisectoral collaboration, which have been previously proposed by various studies conducted in South Asia [77,78].

### Limitations of the Study

While the findings are representative of a large and meaningful sample of regions in Pakistan, there are several limitations to the data that must be considered in the interpretation of our findings. Firstly, the cross-sectional nature of PDHS datasets weakens the study findings as changes over time for the same individual could not be examined. Secondly, the AJK and FATA regions were omitted from the 2017–2018 data so that it was comparable to the earlier survey which did not include these regions. Therefore, our findings are only applicable to the included regions. The AJK and FATA regions have been excluded by all national and regional surveys conducted before 2017 owing to conflicts, military restrictions, and security reasons [29]. The improvement in the current security situation has opened avenues for assessing and implementing health interventions in these restricted areas of Pakistan. The preliminary analysis of the 2017–2018 PDHS showed 55% malnutrition in FATA and 30.8% in the AJK region (Supplementary File S4). This reflects a dire need for intervention in these areas of Pakistan. Thirdly, the datasets used in this study were not able to address the relationship and its impact of various environmental (flooding, famine, food insecurity) and socioeconomic factors (inflation, income, family composition, purchasing power of food, and other necessities) with CFM. Thus, there is a need to include these factors in the health and demographic surveys at a national and regional level. The inclusion of these variables, such as purchasing power, household income, and various environmental factors will provide strong insights into the socioeconomic determinants of CFM [79,80]. Lastly, the nutritional status of children in this study was assessed by simple anthropometric measurements. Though various methods for assessing the nutritional status exist, such as physical examination, biochemical tests, and dietary recall methods, the outcome measures for assessing the nutritional status by these methods were not available in the PDHS datasets.

## 5. Conclusions

In Pakistan, CFM is a major public health problem, and one-third of children aged under five years old are malnourished. Sindh and Baluchistan showed a persistently higher prevalence of CFM, compared with other regions of Pakistan. A significant association of socioeconomic status with various forms of CFM was found, except for the coexistence of underweight with wasting. Improving socioeconomic status may help to alleviate various forms of CFM. Integrating nutrition sensitive and nutrition-specific interventions are effective ways to alleviate malnutrition, including CFM, and thus active engagement of multiple stakeholders, not only from health sectors, but also from education, food and agriculture, economics, and planning and development is essential for combatting issues related to malnutrition.

## Figures and Tables

**Table 1:** Characteristics of the study population—PDHS 2012–2013 and PDHS 2017–2018 datasets.

Variable	Category	PDHS 2012–2013	PDHS 2017–2018	Total	*p*-Value
Wealth index	Poorest	639 (21.7%)	661 (20.5%)	1300 (21.1%)	0.008
	Poorer	583 (19.8%)	745 (23.1%)	1328 (21.5%)	
	Middle	524 (17.8%)	600 (18.6%)	1124 (18.2%)	
	Richer	630 (21.4%)	619 (19.2%)	1249 (20.2%)	
	Richest	571 (19.4%)	596 (18.5%)	1167 (18.9%)	
Sex of child	Male	1488 (50.5%)	1651 (51.3%)	3139 (50.9%)	0.548
	Female	1459 (49.5%)	1570 (48.7%)	3029 (49.1%)	
Child age in months	0 to 11.9 months	542 (18.4%)	619 (19.2%)	1161 (18.8%)	0.053
	12 to 23.9 months	522(17.7%)	647 (20.1%)	1169 (19%)	
	24 to 35.9 months	636 (21.6%)	631 (19.6%)	1267 (20.5%)	
	36 to 47.9 months	613 (20.8%)	673 (20.9%)	1286 (20.8%)	
	48 to 59.9 months	634 (21.5%)	651 (20.2%)	1285 (20.8%)	
Maternal education	No education	1573 (53.4%)	1650 (51.2%)	3223 (52.3%)	<0.001
	Primary	473 (16.1%)	426 (13.2%)	899 (14.6%)	
	Secondary or Higher	901 (30.6%)	1145 (35.5%)	2046 (33.2%)	
Maternal work status	Unemployed	2334 (79.2%)	2816 (87.5%)	5152 (83.5%)	<0.001
	Employed	613 (20.8%)	403 (12.5%)	1016 (16.5%)	
Paternal education	No education	885 (30%)	937 (29.5%)	1822 (29.7%)	0.892
	Primary	439 (14.9%)	476 (15%)	915 (14.9%)	
	Secondary or Higher	1623 (55.1%)	1766 (55.6%)	3389 (55.3%)	
Paternal work status ^¥^*	Unemployed	75 (2.5%)	93 (2.9%)	168 (2.7%)	0.409
	Employed	2872 (97.5%)	3128 (97.1%)	6000 (97.3%)	
Family size	1 to 7 members	1301 (44.1%)	1321 (41%)	2622 (42.5%)	0.013
	8 or more members	1646 (55.9%)	1900 (59%)	3546 (57.5%)	
Source of drinking water *	Improved	2617 (88.8%)	2907 (90.3%)	5524 (89.6%)	0.063
	Unimproved	330 (11.2%)	314 (7.7%)	644 (10.4%)	
Type of toilet *	Improved	2207 (74.9%)	2631 (81.7%)	4839 (78.5%)	<0.001
	Unimproved	740 (25.1%)	589 (18.3%)	1329 (21.5%)	
Housing infrastructure *	Fully constructed.	1336 (45.3%)	1725 (53.6%)	3061 (49.6%)	<0.001
	Semi-constructed	576 (19.5%)	662 (20.6%)	1238 (20.1%)	
	Unconstructed	1035 (35.1%)	834 (25.9%)	1869 (30.3%)	
Region *^¥^	Punjab	920 (31.2%)	839 (26%)	1759 (28.5%)	<0.001
	Sindh	682 (23.1%)	754 (23.4%)	1436 (23.3%)	
	Khyber Pakhtunkhwa	532 (18.1%)	671 (20.8%)	1204 (19.5%)	
	Baluchistan	301 (10.2%)	465 (14.4%)	766(12.4%)	
	Gilgit Baltistan	300 (10.2%)	269 (8.4%)	569 (9.2%)	
	Islamabad	212 (7.2%)	223 (6.9%)	434 (7%)	
Type of place of residence	Urban	1256 (42.6%)	1517 (47.1%)	2773 (45%)	<0.001
	Rural	1691 (57.4%)	1704 (52.9%)	3395 (55%)	
Year of survey	2012–2013	2947 (47.8%)	-	6168 (100%)	-
	2017–2018	-	3221 (52.2%)		

* = Variables were not included for the inferential analysis: ^¥^ = non-uniform distribution between various categories of paternal employment: ICT = Islamabad Capital Territory: GB = Gilgit Baltistan.

**Table 2:** National, provincial, and regional prevalence of various types of malnutrition in children of Pakistan (PDHS 2012–2013 and PDHS 2017–2018).

Year	Pakistan	Punjab	Sindh	KPK	Baluchistan	GB	ICT
	POR (95% CI)	POR (95% CI)	POR (95% CI)	POR (95% CI)	POR (95% CI)	POR (95% CI)	POR (95% CI)
**Malnutrition**							
**2012–2013**	54.4%	44.6%	64.4%	47.7%	86%	55.7%	35.1%
	(52.6 to 56.2%)	(41.3 to 47.8%) *	(60.6 to 67.9%) *	(43.3 to 51.9%) *	(81.6 to 89.7%) *	(49.8 to 61.3%)	(28.6 to 41.9%) *
**2017–2018**	43.3%	30.3%	51.6%	42.8%	64.1%	40.5%	25.6%
	(41.5 to 45%) ^¥^	(27.1 to 33.5%) *^¥^	(47.9 to 55.2%) *^¥^	(38.9 to 46.6%)	(59.5 to 68.4%) *^¥^	(34.6 to 46.6%) ^¥^	(19.9 to 31.8%) *
**Standalone forms of malnutrition**							
**2012–2013**	23.8%	22%	23.3%	22.5%	25.6%	33%	20.4%
	(20.8 to 26.7%)	(19.3 to 24.7%)	(20.1 to 26.6%)	(19 to 26.3%)	(20.7 to 30.9%)	(27.7 to 38.6%) *	(15.1% to 26.4%)
**2017–2018**	21.7%	18.7%	19.6%	25.5%	25.6%	25.7%	16.1%
	(20.3 to 23.2%)	(16.1 to 21.5%)	(16.8 to 22.6%)	(22.2 to 28.9%)	(21.6 to 29.8%)	(20.5 to 31.3%)	(11.5 to 21.6%)
**Coexisting forms of malnutrition**							
**2012–2013**	30.6%	22.6%	41.1%	25.1%	60.5%	22.7%	14.7%
	(27.1 to 34.5%)	(19.9 to 25.4%) *	(37.3 to 44.8%) *	(21.5 to 29%)	(50.1 to 71.4%) *	(18 to 27.8%) *	(10.2 to 20.2%) *
**2017–2018**	21.5%	11.6%	32%	17.3%	38.5%	14.9%	9.4%
	(20.1 to 23%) ^¥^	(9.4 to 13.9%) *^¥^	(28.6 to 35.4%) *^¥^	(14.5 to 20.3%) ^¥^	(34 to 43.1%) *^¥^	(10.8 to 19.6%)	(5.9 to 14%) *
**Coexisting forms of undernutrition**							
**2012–2013**	24.5%	21.5%	37.1%	22.1%	30.9%	11.7%	11.8%
	(21.8 to 27.4%)	(18.9 to 24.3%)	(33.4 to 40.8%) *	(18.6 to 25.9%)	(25.7 to 36.4%) *	(8.2 to 15.8%) *	(7.8 to 16.9%) *
**2017–2018**	20.1%	11.2%	30.1%	15.9%	36.3%	11.9%	8.8%
	(18.7 to 21.5%) ^¥^	(9.1 to 13.5%) *^¥^	(26.8 to 33.5%) *^¥^	(13.2 to 18.9%) ^¥^	(31.9 to 40.9%) *	(8.2 to 16.3%) *	(5.3 to 12.9%) *
**Coexisting forms of overnutrition (Paradox)**							
**2012–2013**	6.1%	1.1%	4%	3%	29.6%	11%	2.8%
	(5.3 to 7.1%)	(0.5 to 1.9%) *	(2.6 to 5.7%)	(1.7 to 4.8%)	(24.4 to 35.1%) *	(7.6 to 15.1%) *	(1.1 to 6.1%)
**2017–2018**	1.4%	0.4%	1.9%	1.3%	2.2%	3.0%	0.9%
	(1 to 1.9%) ^¥^	(0.01 to 0.1%) *	(1 to 3.1%)	(0.6 to 2.5%)	(1 to 3.9%) ^¥^	(1.2 to 5.7%) ^¥^	(0.1 to 3.2%)
**Coexistence of underweight with stunting**							
**2012–2013**	17.2%	16.2%	26.5%	14.6%	19.9%	8.3%	6.2%
	(15.8 to 18.6%)	(13.8 to 18.7%)	(23.2 to 30.2%) *	(11.7 to 17.9%)	(15.5 to 24.9%)	(5.4 to 12.1%) *	(3.3 to 10.3%) *
**2017–2018**	14.3%	8.6%	22.7%	11.6%	21.1%	10.8%	5.4%
	(13.1 to 15.5%) ^¥^	(6.7 to 10.7%) *^¥^	(19.7 to 25.8%) *	(9.2 to 14.2%)	(17.4 to 25.1%) *	(7.3 to 15.1%)	(2.8 to 9.2%) *
**Coexistence of underweight with wasting**							
**2012–2013**	2.9%	2.7%	3.7%	3%	2%	2.3%	3.3%
	(2.3 to 3.6%)	(1.7 to 3.9%)	(2.3 to 5.3%)	(1.7 to 4.8%)	(0.7 to 4.2%)	(0.9 to 4.7%)	(1.3 to 6.7%)
**2017–2018**	3.1%	1.1%	3.3%	3.1%	8.8%	0% ^¥^	2.2%
	(2.5 to 3.8%)	(0.5 to 2%) *	(2.1 to 4.8%)	(1.9 to 4.7%)	(6.4 to 11.7%) *		(0.7 to 5.1%)
**Coexistence of underweight with stunting and wasting**							
**2012–2013**	4.4%	2.6%	6.9%	4.5%	9%	1%	2.4%
	(3.7 to 5.2%)	(1.6 to 3.8%)	(5.1 to 9.1%)	(2.9 to 6.6%)	(5.9 to 12.7%) *	(0.2 to 2.8%) *	(0.7 to 5.4%)
**2017–2018**	2.7%	1.5%	4.1%	1.2%	6.5%	1.1%	0.9%
	(2.1 to 3.3%) ^¥^	(0.8 to 2.6%)	(2.8 to 5.7%)	(0.5 to 2.3%) ^¥^	(4.3 to 9.1%) *^¥^	(0.2 to 3.2%)	(0.1 to 3.2%)

POR (Prevalence Odds Ratio) = Prevalence odds ratio: CI = Confidence interval: * = The regional prevalence is significantly different from that of national prevalence: ^¥^ = The prevalence of malnutrition in 2017–2018 was significantly different from 2012–2013: KPK = Khyber Pakhtunkhwa: GB = Gilgit Baltistan: ICT = Islamabad Capital Territory.

**Table 3:** Assessing the interaction of different covariates with various forms of coexisting forms of malnutrition across two survey periods.

Year Interaction with Variables	Year * Categories	Coexistence of Underweight with Wasting ^¥^	Coexistence of Underweight with Stunting ^¥^	Coexistence of Underweight with Wasting and Stunting Both ^¥^	Coexistence of Stunting with Overweight/Obesity ^¥¥^
		OR (95% CI)	OR (95% CI)	OR (95% CI)	OR (95% CI)
Year * Socioeconomic status	Year * Poorest	Ref	Ref	Ref	Ref
	Year * Poorer	2.08 (0.40 to 10.67)	0.83 (0.19 to 3.64)	2.77 (0.56 to 13.63)	3.06 (0.95 to 9.83)
	Year * Middle	0.76 (0.13 to 4.41)	0.42 (0.09 to 2.02)	0.69 (0.12 to 3.98)	2.66 (0.77 to 9.12)
	Year * Richer	2.17 (0.29 to 16.01)	0.70 (0.11 to 4.11)	1.17 (0.17 to 7.94)	5.68 (1.67 to 19.30) *
	Year * Richest	0.30 (0.05 to 1.79)	0.53 (0.12 to 2.40)	0.32 (0.03 to 2.66)	5.27 (1.72 to 16.11) *
Year * Sex	Year * Male	Ref	Ref	Ref	Ref
	Year * Female	1.09 (0.35 to 3.31)	0.76 (0.28 to 2.04)	0.61 (0.20 to 1.84)	1.25 (0.62 to 2.51)
Year * Age	Year * 0–11 mo	Ref	Ref	Ref	Ref
	Year * 12–23 mo	1.88 (0.27 to 12.67)	5.65 (0.93 to 34.37)	2.15 (0.31 to 15.28)	0.63 (0.16 to 2.40)
	Year * 24–35 mo	2.15 (0.38 to 12.14)	1.91 (0.39 to 9.22)	0.72 (0.12 to 4.37)	1.63 (0.55 to 4.74)
	Year * 36–47 mo	4.66 (0.68 to 31.90)	9.80 (1.66 to 57.65) *	3.92 (0.55 to 27.57)	0.20 (0.05 to 0.78) *
	Year * 48–59 mo	1.53 (0.30 to 7.77)	2.42 (0.57 to 10.19)	1.77 (0.33 to 9.53)	0.43 (0.13 to 1.37)
Year * Maternal education	Year * No education	Ref	Ref	Ref	Ref
	Year * Primary	1.65 (0.29 to 9.38)	1.07 (0.23 to 4.86)	1.00 (0.16 to 6.25)	0.96 (0.29 to 3.22)
	Year * Secondary or higher	0.48 (0.13 to 1.75)	0.54 (0.17 to 1.65)	0.36 (0.10 to 1.32)	2.29 (1.07 to 4.86) *
Year * Maternal working status	Year * No	Ref	Ref	Ref	Ref
	Year * Yes	0.46 (0.11 to 1.81)	0.59 (0.19 to 1.86)	0.63 (0.17 to 2.32)	0.27 (0.03 to 2.11)
Year * Paternal education	Year * No education	Ref	Ref	Ref	Ref
	Year * Primary	1.04 (0.13 to 8.08)	1.55 (0.24 to 10.04)	1.81 (0.24 to 13.14)	0.61 (0.14 to 2.58)
	Year * Secondary or higher	0.68 (0.19 to 2.37)	0.71 (0.23 to 2.14)	0.82 (0.24 to 2.81)	2.32 (0.99 to 5.41)
Year * Paternal working status	Year * No	Ref	Ref	Ref	Ref
	Year * Yes	1.81 (0.00 to inf)	1.42 (0.00 to inf)	1.01 (0.00 to inf)	1.91 × 10^6^ (1.92 × 10^−286^ to 1.89 × 10^298^)
Year * Family size	Year * 1 to 7 members	Ref	Ref	Ref	Ref
	Year * 8 or more members	1.20 (0.39 to 3.67)	1.34 (0.50 to 3.60)	1.48 (0.49 to 4.47)	0.64 (0.32 to 1.31)
Year * Type of place of residence	Year * Rural	Ref	Ref	Ref	Ref
	Year * Urban	0.71 (0.22 to 2.25)	0.36 (0.13 to 1.02)	0.45 (0.14 to 1.41)	1.69 (0.83 to 3.41)

Asterix sign (*) showed a significant interaction of covariate across two survey periods with various forms of Coexisting forms of Malnutrition (CFM). ^¥^ = The reference category for assessing the determinants of coexistence of underweight with wasting, the coexistence of underweight with stunting, and coexistence of underweight with wasting and stunting both was underweight. ^¥¥^ = The reference category for assessing the determinants of coexistence of stunting with overweight/obesity was stunting.

**Table 4:** Assessing the determinants of various types of coexisting forms of malnutrition in children under five years of age.

Variable	Categories	Coexistence of Underweight with Wasting ^¥^		Coexistence of Underweight with Stunting ^¥^		Coexistence of Underweight with Wasting and Stunting Both ^¥^		Coexistence of Stunting with Overweight/Obesity ^¥¥^	
		Unadjusted Odds (95% CI)	Adjusted Odds (95% CI) ^1^	Unadjusted Odds (95% CI)	Adjusted Odds (95% CI) ^2^	Unadjusted Odds (95% CI)	Adjusted Odds (95% CI) ^3^	Unadjusted Odds (95% CI)	Adjusted Odds (95% CI) ^4^
**Socioeconomic status**	**Poorest**	Ref	Ref	Ref	Ref	Ref	Ref	Ref	Ref
	**Poorer**	1.15 (0.52 to 2.54)	0.99 (0.43 to 2.23)	0.60 (0.29 to 1.22)	0.66 (0.31 to 1.37)	0.61 (0.28 to 1.32)	0.55 (0.23 to 1.27)	0.59 (0.39 to 0.91) *	0.50 (0.32 to 0.79) *
	**Middle**	0.77 (0.32 to 1.86)	0.68 (0.28 to 1.67)	0.45 (0.21 to 0.98) *	0.47 (0.21 to 1.05)	0.41 (0.17 to 0.97) *	0.34 (0.13 to 0.87) *	0.58 (0.37 to 0.91) *	0.48 (0.29 to 0.78) *
	**Richer**	0.91 (0.34 to 2.36)	0.72 (0.26 to 1.93)	0.72 (0.31 to 1.69)	0.67 (0.28 to 1.62)	0.78 (0.31 to 1.94)	0.78 (0.29 to 2.11)	0.59 (0.37 to 0.95) *	0.39 (0.22 to 0.66) *
	**Richest**	0.45 (0.19 to 1.05)	0.36 (0.15 to 0.87) *	0.16 (0.08 to 0.34) *	0.18 (0.08 to 0.41) *	0.13 (0.05 to 0.32) *	0.10 (0.04 to 0.27) *	1.75 (1.14 to 2.67) *	1.18 (0.70 to 1.99)
**Sex**	**Male**	Ref		Ref		Ref		Ref	
	**Female**	1.25 (0.72 to 2.15)		1.11 (0.68 to 1.80)		0.74 (0.43 to 1.28)		1.11 (0.83 to 1.48)	
**Age**	**0–11 mo**	Ref		Ref	Ref	Ref	Ref	Ref	Ref
	**12–23 mo**	2.33 (1.00 to 5.42) *		5.59 (2.55 to 12.25) *	5.08 (2.28 to 11.29) *	8.47 (3.55 to 20.23) *	9.45 (3.75 to 23.78) *	0.11 (0.06 to 0.21) *	0.11 (0.06 to 0.21) *
	**24–35 mo**	2.08 (0.88 to 4.89)		9.42 (4.33 to 20.50) *	8.82 (4.01 to 19.43) *	5.88 (2.42 to 14.25) *	5.64 (2.22 to 14.28) *	0.16 (0.09 to 0.26) *	0.14 (0.08 to 0.25) *
	**36–47 mo**	1.60 (0.69 to 3.70)		8.90 (4.21 to 18.82) *	8.50 (3.96 to 18.21) *	6.03 (2.56 to 14.17) *	8.53 (3.36 to 21.61) *	0.18 (0.11 to 0.29) *	0.16 (0.09 to 0.28) *
	**48–59 mo**	1.29 (0.57 to 2.87)		6.97 (3.44 to 14.13) *	6.22 (3.02 to 12.79) *	3.79 (1.65 to 8.72) *	4.52 (1.84 to 11.08) *	0.24 (0.14 to 0.39) *	0.22 (0.13 to 0.38) *
**Maternal education**	**No education**	Ref		Ref		Ref		Ref	
	**Primary**	0.45 (0.20 to 0.99) *		0.57 (0.29 to 1.12)		0.28 (0.12 to 0.64) *		1.02 (0.66 to 1.57)	
	**Secondary and higher**	0.47 (0.25 to 0.89) *		0.32 (0.19 to 0.56) *		0.36 (0.19 to 0.68) *		1.33 (0.96 to 1.84)	
**Maternal working status**	**No**	Ref	Ref	Ref		Ref		Ref	Ref
	**Yes**	0.55 (0.28 to 1.07)	0.47 (0.23 to 0.95) *	0.95 (0.55 to 1.67)		0.82 (0.44 to 1.55)		0.62 (0.40 to 0.95) *	0.49 (0.31 to 0.79) *
**Paternal education**	**No education**	Ref		Ref		Ref		Ref	
	**Primary**	0.97 (0.38 to 2.49)		1.06 (0.46 to 2.44)		1.10 (0.45 to 2.72)		0.81 (0.49 to 1.32)	
	**Secondary and higher**	0.67 (0.36 to 1.25)		0.46 (0.26 to 0.78) *		0.47 (0.25 to 0.86) *		1.43 (1.03 to 1.98) *	
**Paternal working status ∞**	**No**							Ref	
	**Yes**							0.51 (0.25 to 1.05)	
**Family size**	**1 to 7 members**	Ref		Ref		Ref		Ref	
	**8 or more members**	1.14 (0.66 to 1.98)		1.14 (0.70 to 1.85)		1.17 (0.68 to 2.01)		1.20 (0.89 to 1.61)	
**Type of place of residence**	**Rural**	Ref		Ref		Ref		Ref	Ref
	**Urban**	0.80 (0.46 to 1.38)		0.52 (0.32 to 0.85) *		0.92 (0.53 to 1.57)		1.41 (1.06 to 1.88) *	1.50 (1.04 to 2.16) *
**Survey year**	**2012–2013**	Ref		Ref		Ref		Ref	Ref
	**2017–2018**	1.85 (1.06 to 3.21)		1.43 (0.87 to 2.33)		1.05 (0.61 to 1.82)		0.25 (0.17 to 0.35) *	0.22 (0.15 to 0.32) *

^¥^ = The reference category for assessing the determinants of coexistence of underweight with wasting, the coexistence of underweight with stunting, and coexistence of underweight with wasting and stunting both was underweight. ^¥¥^ = The reference category for assessing the determinants of coexistence of stunting with overweight/obesity was stunting ∞ = Paternal employment status showed confounding effect for all outcomes, except for the coexistence of stunting with overweight/obesity. ^1^ = Adjusted for socioeconomic status, and maternal working status. ^2^ = Adjusted for socioeconomic status, and child age. ^3^ = Adjusted for socioeconomic status, and child age. ^4^ = Adjusted for socioeconomic status, child age, maternal employment status, type of place of residence, and survey year.

## Data Availability

Not applicable.

## Supplementary Materials

The following are available online at https://www.mdpi.com/article/10.3390/nu13124566/s1, Supplementary File S1: Description of the process for creating various categories of the Coexisting forms of malnutrition. Table S1: Nutritional outcome and different coding, Supplementary File S2: Graphically comparing the national and regional prevalence of various types of malnutrition in children of Pakistan across two survey periods. Figure S1: National, provincial, and regional prevalence of various types of malnutrition in children of Pakistan (PDHS 2012–2013 and PDHS 2017–2018). Supplementary File S3: It has four tables, and each table represents the determinants of each type of CFM, such as Table S3A examined the determinants of coexistence of underweight with wasting for each survey year: 2012–2013 and 2017–2018. However, Table S3B, Table S3C, and Table S3D showed the determinants of coexistence of underweight with stunting, the coexistence of underweight with both wasting and stunting, and coexistence of stunting with overweight/obesity, respectively. Supplementary File S4: Representing the prevalence of various forms of malnutrition in the AJK and FATA region. While the data of these two regions were not included in the study for analysis.
