# Supplementary material for: Prevalence, Trends, and Socioeconomic Determinants of Coexisting Forms of Malnutrition Amongst Children under Five Years of Age in Pakistan
Source: Nutrients. 2021 Dec 20;13(12):4566. doi: 10.3390/nu13124566 (PMC8707290; doi:10.3390/nu13124566)
Supplement: Supplementary file 1 [file nutrients-13-04566-s001.zip › nutrients-1437015 - supplementary.pdf]

Supplementary file S1

A numeric scoring system was assigned for assessing the various types of nutritional status. Initially, the z-scores for each anthropometric index were assessed and based on the z-score value we determined the child nutritional. For Length/Height for Age (LAZ/HAZ) z-scores, and Weight for Age (WAZ), two categories for nutritional status were created. However, for Weight for Length/Height (WLZ/WHZ), three categories were created: Wasting, Normal, and Overweight/Obese. After determining the child nutritional status, numeric codes were assigned to each category. A numeric code of '0' was assigned to a child with Normal nutritional status. While the code assigned for wasting, stunting, underweight, and overweight/obesity were '1', '2', '5' and '10', respectively. A new variable was created, and this variable depicted overall nutritional status of each child. This new variable represents the numeric coding sum of HAZ, WAZ and WHZ. The new variable has 9 categories, and each category represent a type of nutritional status. The scoring/coding for each nutritional outcome is presented in Supplementary table 1.

Table S1: Nutritional outcomes and their coding

**Numeric code**	**Nutritional outcome**
0	Normal
1	Wasting
2	Stunting
5	Underweight
6	Coexistence of underweight with wasting
7	Coexistence of underweight with stunting
8	Coexistence of underweight with wasting and stunting both
10	Overweight/obesity
12	Paradox

Supplementary file S2

Figure S1: National, provincial, and regional prevalence of various types of malnutrition in children of Pakistan (PDHS-2012-2013 and PDHS-2017-2018).

<table>
<tr><td>Survey year</td><td>PDHS 2012-2013</td><td>PDHS 2017-2018</td></tr>
<tr><td>Malnutrition</td><td>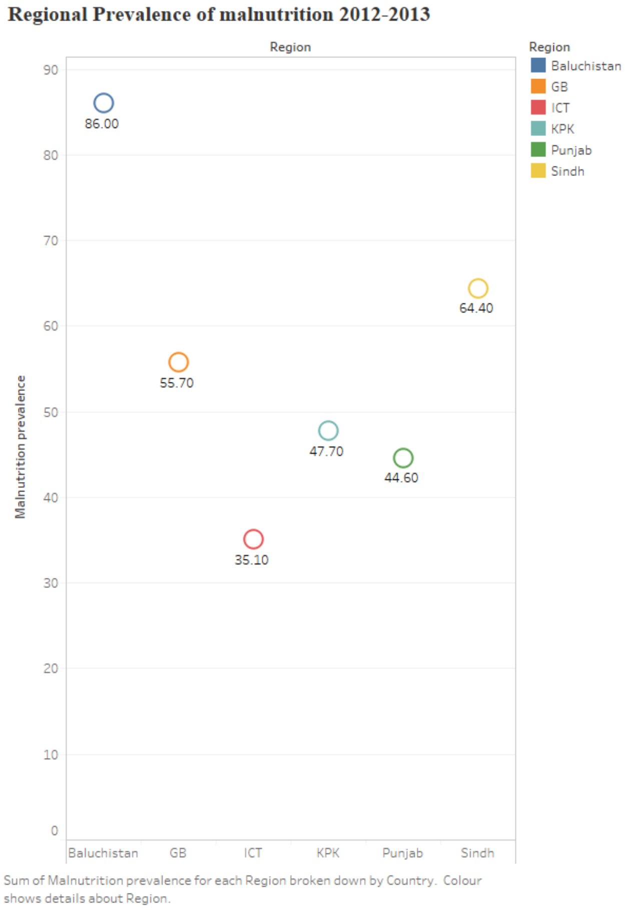
</td><td>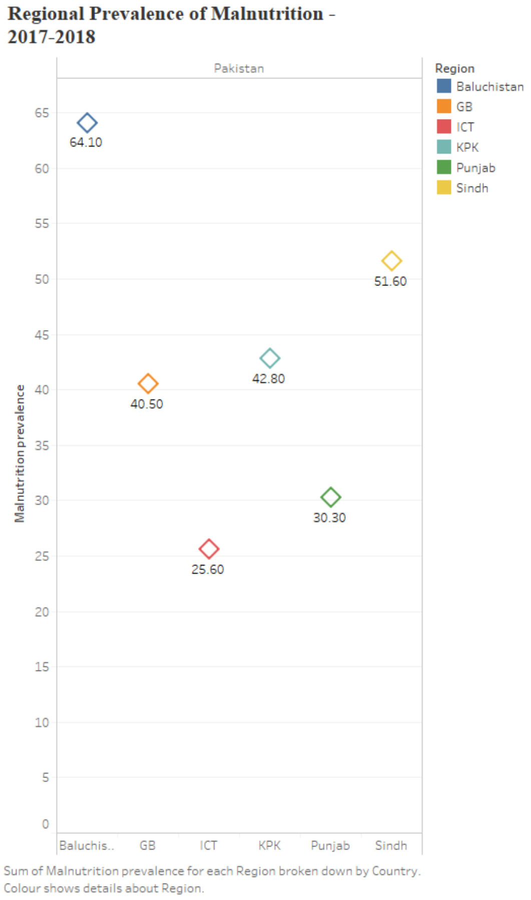
</td></tr>
</table>

Standalone forms of malnutrition	**Regional Prevalence of Standalone forms of Malnutrition 2012-2013**<br>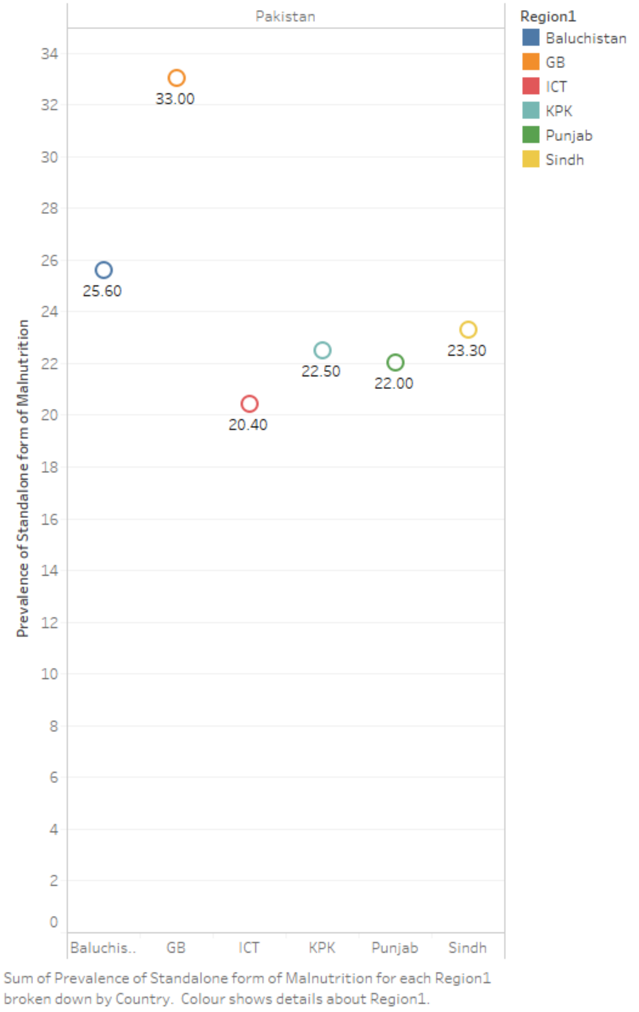	**Regional Prevalence of Standalone forms of Malnutrition - 2017-2018**<br>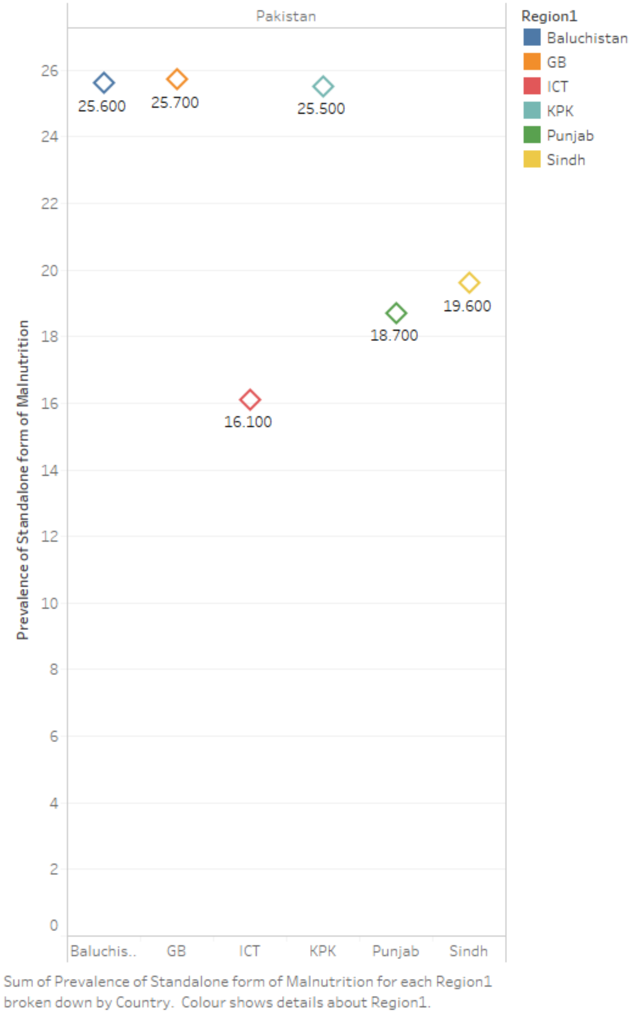

Coexisting forms of malnutrition

Regional Prevalence of Coexisting forms of Malnutrition 2012-2013

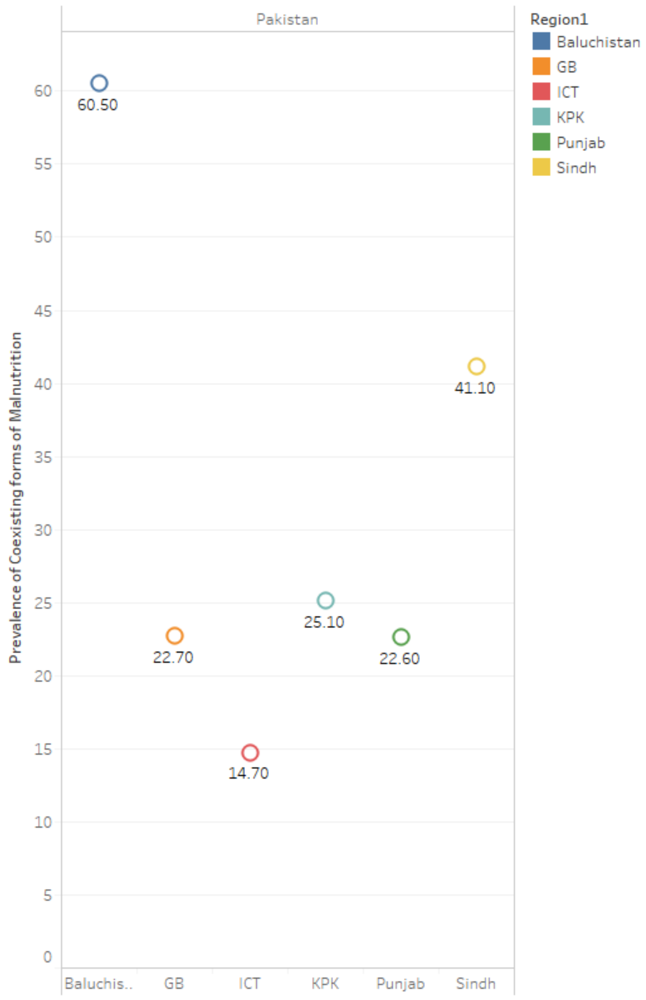

Sum of Prevalence of Coexisting forms of Malnutrition for each Region1 broken down by Country. Colour shows details about Region1.

Regional Prevalence of Coexisting forms of Malnutrition - 2017-2018

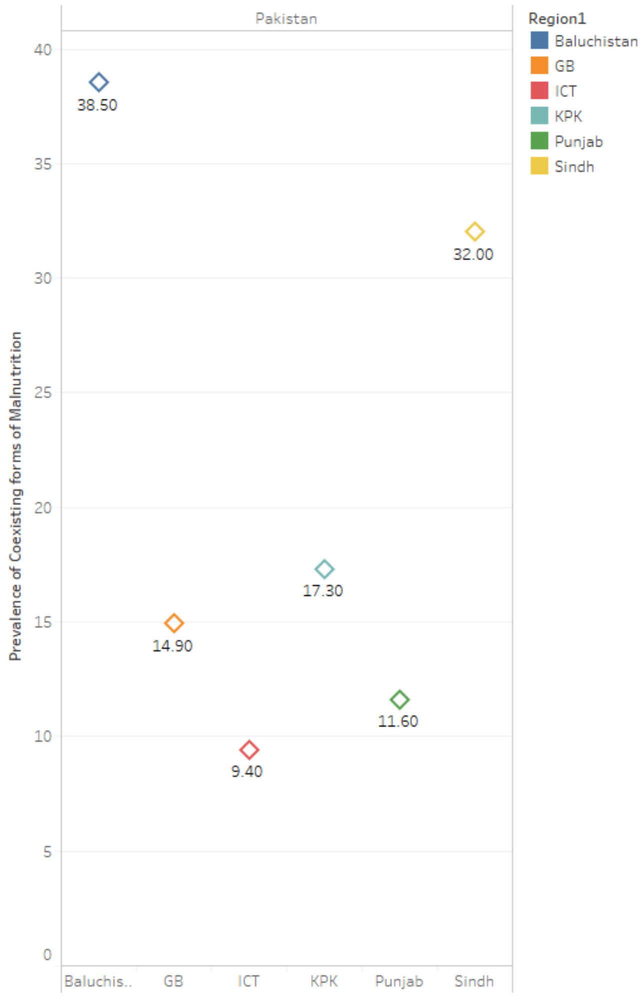

Sum of Prevalence of Coexisting forms of Malnutrition for each Region1 broken down by Country. Colour shows details about Region1.

**Nutritional paradox**	**Regional Prevalence of Nutritional Paradox-2012-2013** 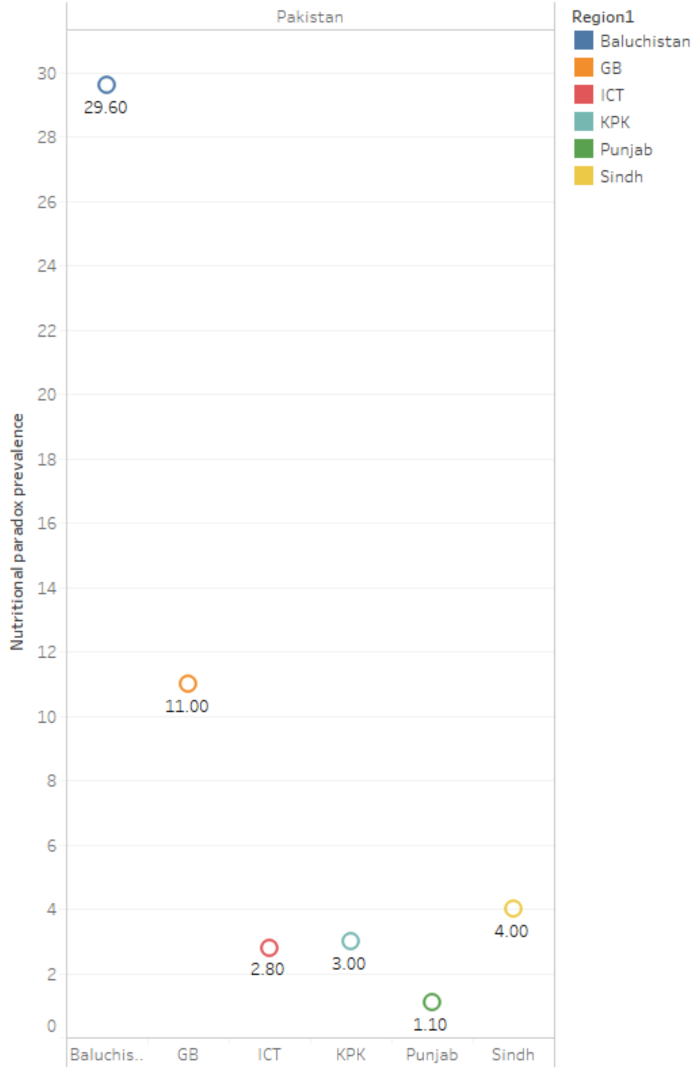 Sum of Nutritional paradox prevalence for each Region1 broken down by Country. Colour shows details about Region1.	**Regional Prevalence of Nutritional Paradox - 2017-2018** 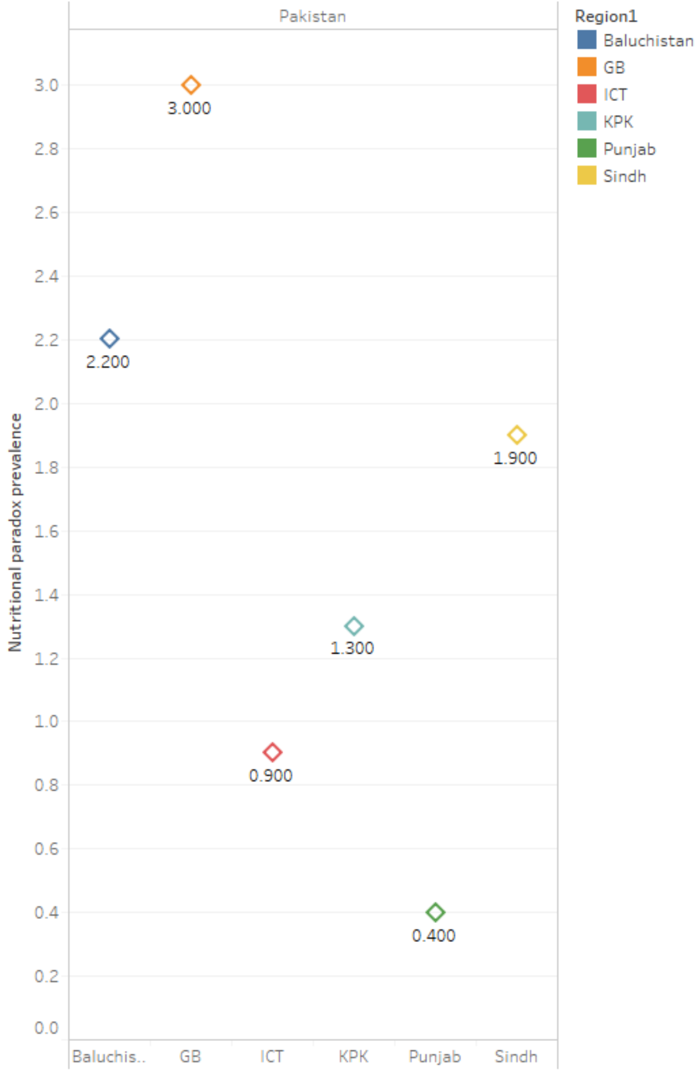 Sum of Nutritional paradox prevalence for each Region1 broken down by Country. Colour shows details about Region1.

Coexistence of undernutrition

Regional Prevalence of Coexisting forms of undernutrition- 2012-2013

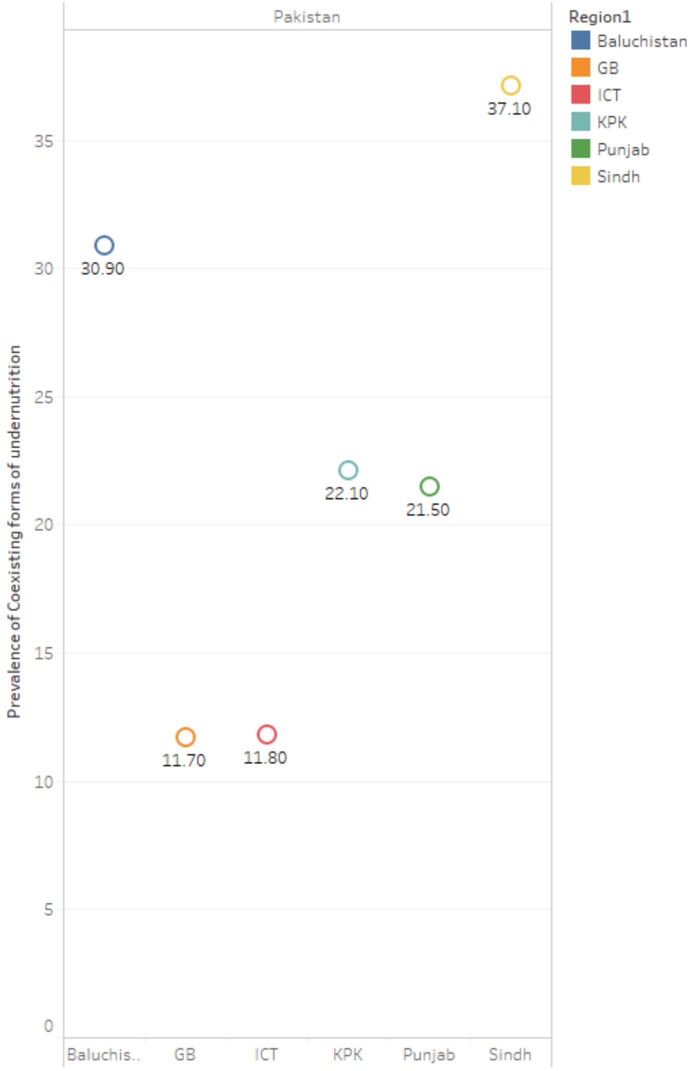

Sum of Prevalence of Coexisting forms of undernutrition for each Region1 broken down by Country. Colour shows details about Region1.

Regional Prevalence of Coexisting forms of undernutrition - 2017-2018

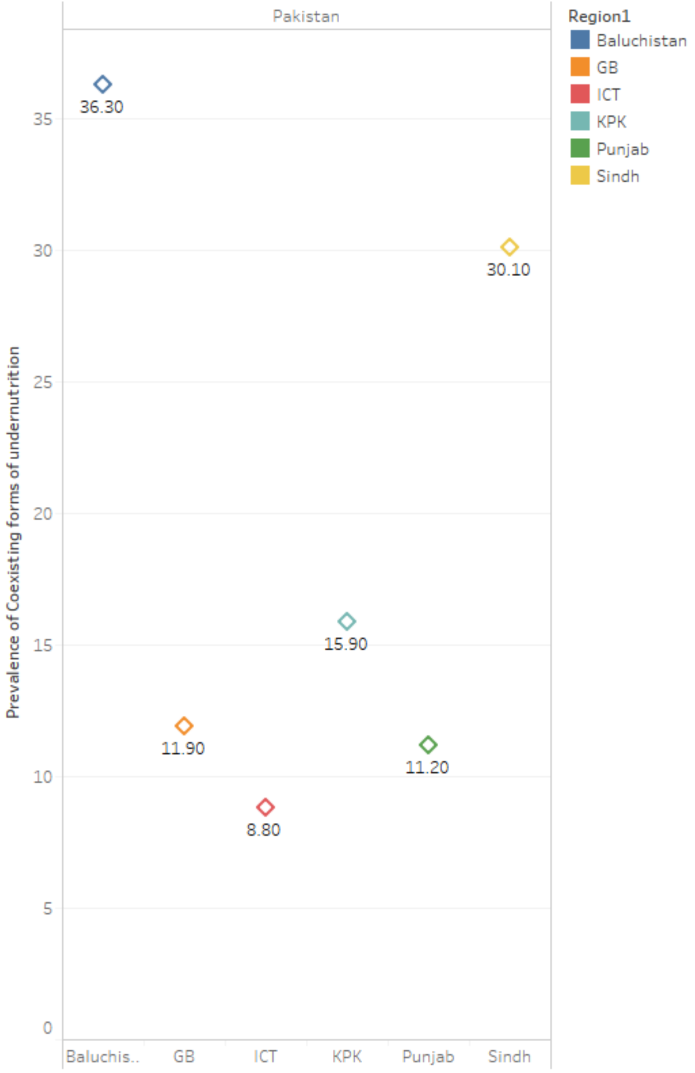

Sum of Prevalence of Coexisting forms of undernutrition for each Region1 broken down by Country. Colour shows details about Region1.

Coexistence of underweight with wasting	**Regional Prevalence of Coexistence of underweight with wasting- 2012-2013** 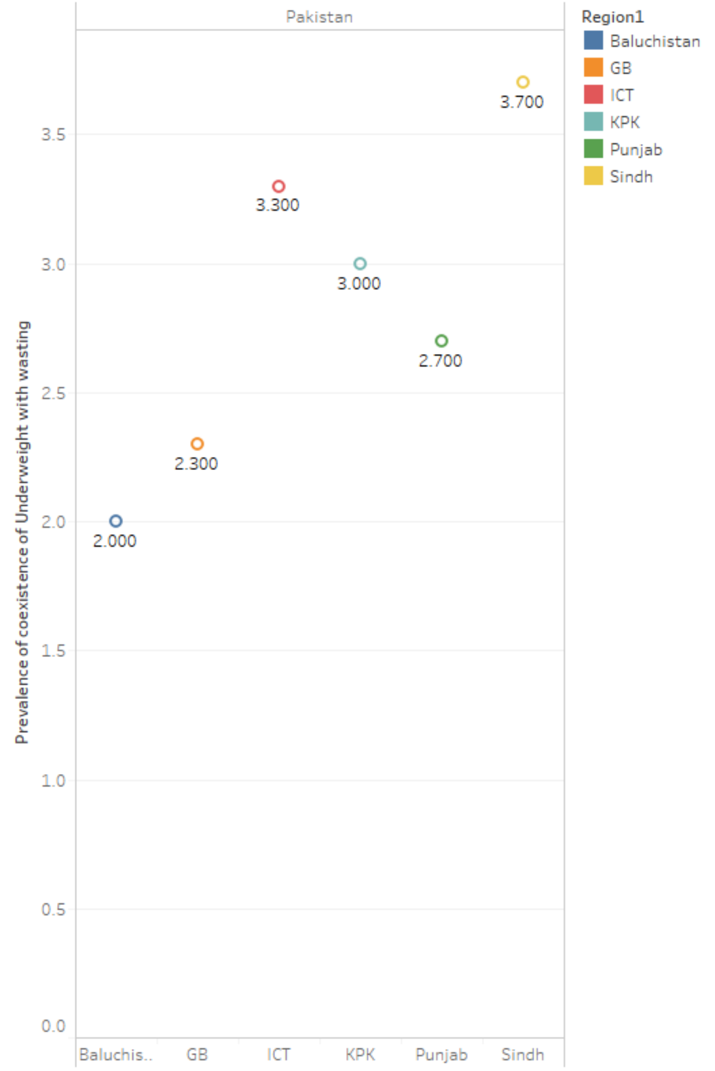	**Regional Prevalence of Coexistence of underweight with wasting - 2017-2018** 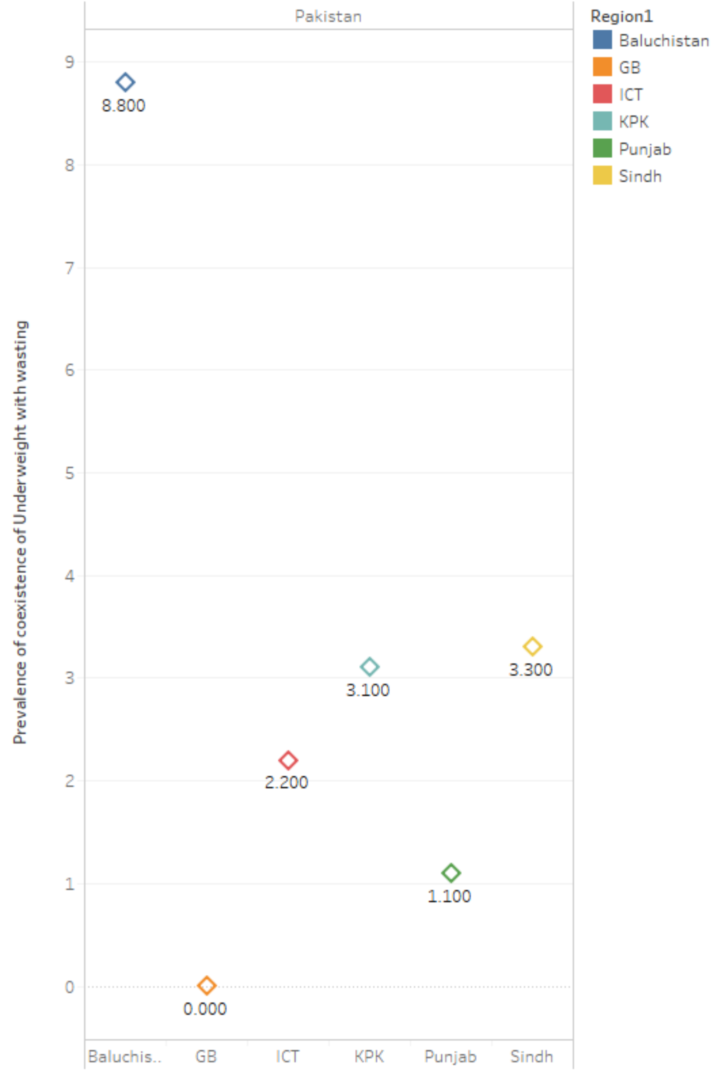

Sum of Prevalence of coexistence of Underweight with wasting for each Region1 broken down by Country. Colour shows details about Region1.

Sum of Prevalence of coexistence of Underweight with wasting for each Region1 broken down by Country. Colour shows details about Region1.

Coexistence of underweight with stunting

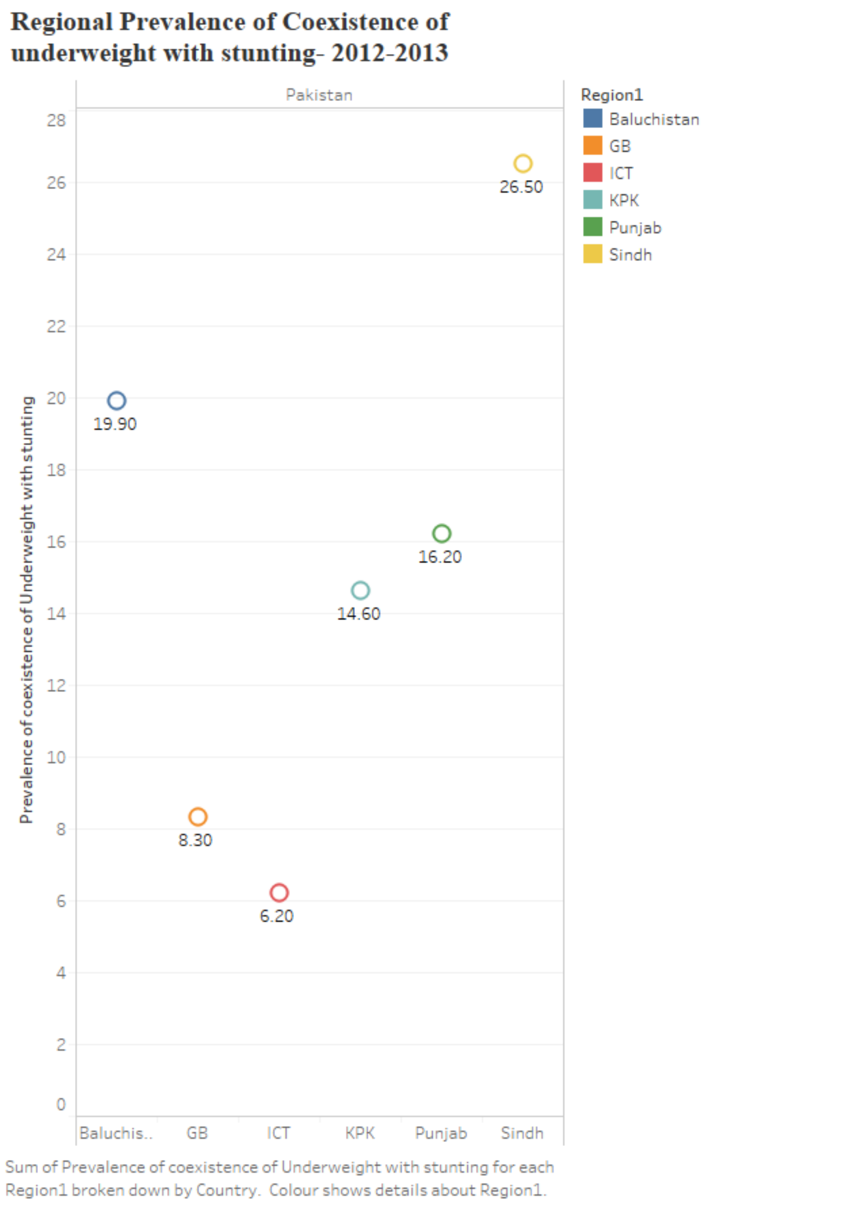

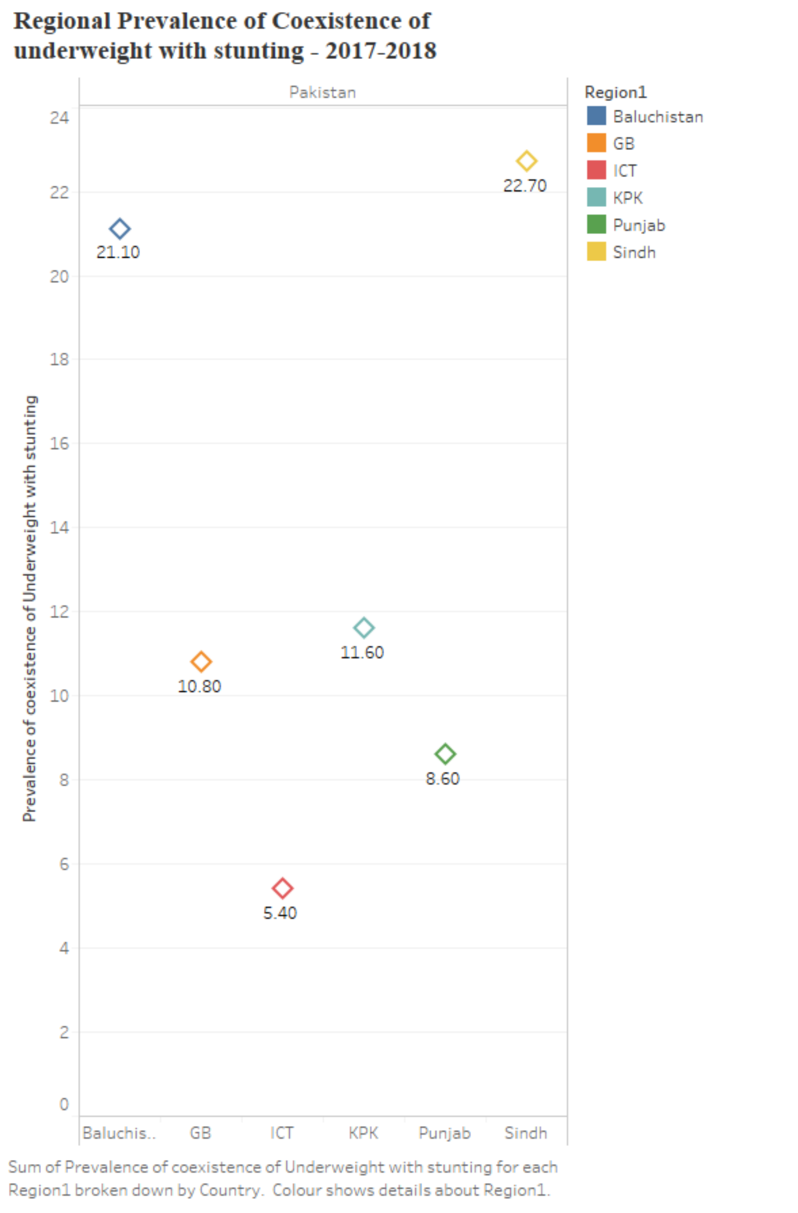

Coexistence of underweight with wasting and stunting

Regional Prevalence of Coexistence of underweight with wasting and stunting-2012-2013

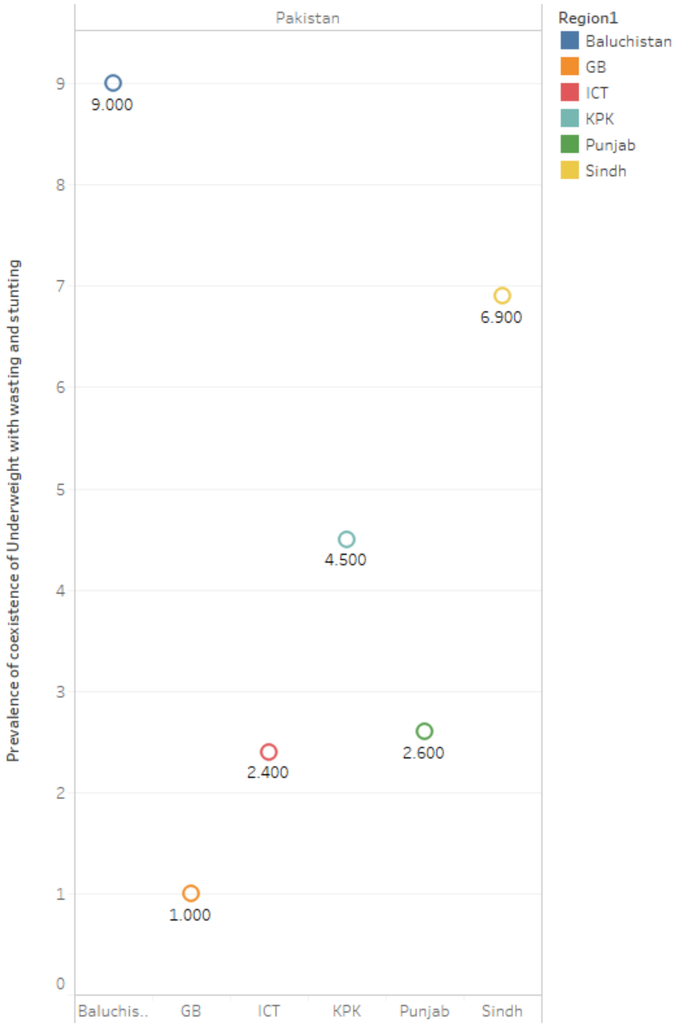

Sum of Prevalence of coexistence of Underweight with wasting and stunting for each Region1 broken down by Country. Colour shows details about Region1.

Regional Prevalence of Coexistence of underweight with wasting and stunting-2017-2018

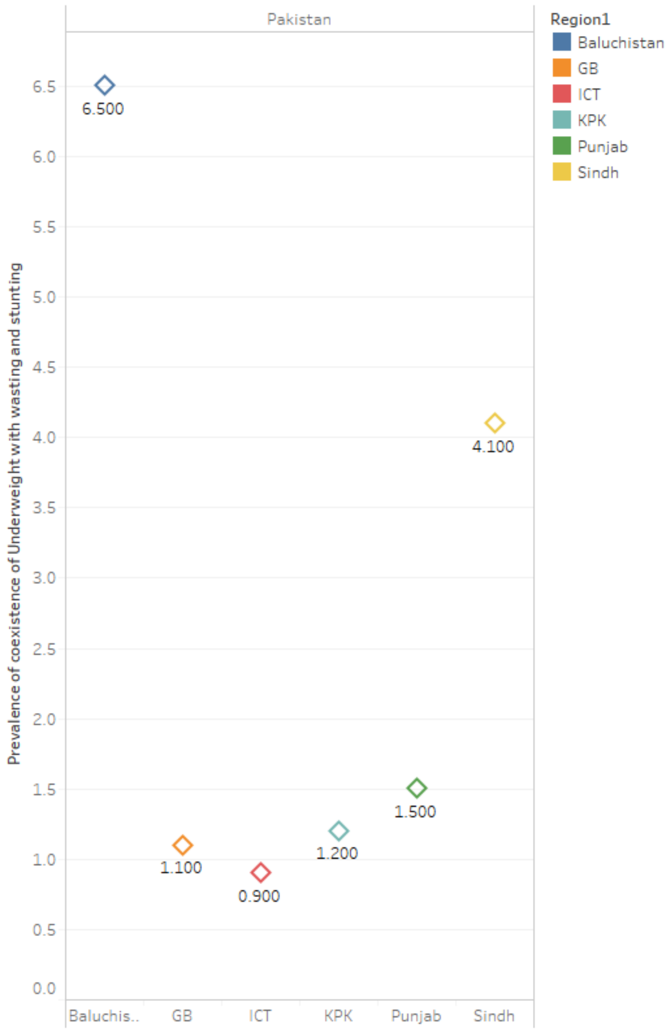

Sum of Prevalence of coexistence of Underweight with wasting and stunting for each Region1 broken down by Country. Colour shows details about Region1.

Supplementary file S3

Table S3. A: Assessing the determinants of coexistence of underweight with wasting (underweight as reference category):

**Variables**	Categories	PDHS 2012-2013		PDHS 2017-2018	
		Unadjusted Odds (95% CI)	Adjusted odds [1] (95% CI)	Unadjusted Odds (95% CI)	Adjusted odds 2 (95% CI)
**Socioeconomic status**	Poorest	Ref	Ref	Ref	Ref
	Poorer	0.87 (0.26 to 2.87)	0.87 (0.26 to 2.87)	1.62 (0.47 to 5.61)	1.45 (0.40 to 5.17)
	Middle	0.78 (0.26 to 2.26)	0.78 (0.26 to 2.26)	0.66 (0.18 to 2.43)	0.63 (0.16 to 2.41)
	Richer	0.61 (0.16 to 2.23)	0.61 (0.16 to 2.23)	1.33 (0.29 to 6.11)	1.04 (0.21 to 5.01)
	Richest	0.71 (0.24 to 2.07)	0.71 (0.24 to 2.07)	0.21 (0.05 to 0.87) *	0.17 (0.04 to 0.74)
Sex	Male	Ref		Ref	
	Female	0.87 (0.42 to 1.81)		1.24 (0.53 to 2.88)	
Age	0-11 mo	Ref		Ref	
	12-23 mo	0.50 (0.17 to 1.42)		3.75 (0.75 to 18.58)	
	24-35 mo	0.74 (0.22 to 2.53)		2.88 (0.84 to 9.83)	
	36-47 mo	1.09 (0.37 to 3.23)		4.25 (0.86 to 20.86)	-
	48-59 mo	0.96 (0.31 to 2.92)		1.60 (0.49 to 5.21)	
Maternal education	No education	Ref		Ref	
	Primary	2.52 (0.92 to 6.86)		0.65 (0.15 to 2.70)	
	Secondary or higher	1.54 (0.63 to 3.76)	-	0.31 (0.12 to 0.79) *	
Maternal working status	No	Ref		Ref	Ref
	Yes	1.25 (0.53 to 2.96)		0.36 (0.12 to 1.06)	0.28 (0.08 to 0.93) *
Paternal education	No education	Ref		Ref	
	Primary	0.92 (0.28 to 2.94)		1.12 (0.21 to 6.10)	
	Secondary or higher	1.22 (0.53 to 2.78)		0.55 (0.22 to 1.42)	
Paternal working status	No	Ref		Ref	
	Yes	1.10e+6 (0.00 to inf)		2.24e+6 (0.00 to inf)	
Family size	1 to 7 members	Ref		Ref	

	8 or more members	0.94 (0.45 to 1.97)		0.78 (0.34 to 1.83)	
Type of place of residence	Rural	Ref		Ref	
	Urban	1.24 (0.58 to 2.68)		0.56 (0.23 to 1.35)	

∞ = The reference category for assessing the determinants of coexistence of underweight with wasting was underweight.

1 = Adjusted for socioeconomic status.

2 = Adjusted for socioeconomic status, and maternal work status.

B: Assessing the determinants of coexistence of underweight with stunting (underweight as reference category):

**Variables**	Categories	PDHS 2012-2013		PDHS 2017-2018	
		Unadjusted Odds (95% CI)	Adjusted odds [1] (95% CI)	Unadjusted Odds (95% CI)	Adjusted odds 2 (95% CI)
**Socioeconomic status**	Poorest	Ref	Ref	Ref	Ref
	Poorer	0.67 (0.27 to 1.68)	0.68 (0.27 to 1.74)	0.56 (0.17 to 1.80)	0.72 (0.21 to 2.57)
	Middle	0.68 (0.24 to 1.89)	0.72 (0.25 to 2.05)	0.29 (0.09 to 0.93) *	0.38 (0.09 to 1.54)
	Richer	0.88 (0.31 to 2.57)	0.85 (0.29 to 2.53)	0.62 (0.15 to 2.56)	1.01 (0.19 to 5.44)
	Richest	0.22 (0.08 to 0.57) *	0.23 (0.08 to 0.62) *	0.12 (0.03 to 0.38) *	0.36 (0.07 to 1.78)
Sex	Male	Ref		Ref	
	Female	1.24 (0.66 to 2.30)		0.94 (0.43 to 2.02)	
Age	0-11 mo	Ref	Ref	Ref	Ref
	12-23 mo	3.02 (1.18 to 7.73) *	2.78 (1.07 to 7.24) *	17.07 (3.66 to 79.51) *	23.09 (4.43 to 120.11) *
	24-35 mo	7.29 (2.57 to 20.67) *	7.21 (2.51 to 20.67) *	13.97 (4.31 to 45.30) *	14.48 (4.14 to 50.66) *
	36-47 mo	3.77 (1.54 to 9.24) *	3.65 (1.46 to 9.12) *	36.98 (8.02 to 170.53) *	45.57 (9.24 to 224.56) *
	48-59 mo	4.86 (1.91 to 12.33) *	4.50 (1.75 to 11.57) *	11.79 (3.96 to 35.12) *	12.61 (3.78 to 42.04) *
Maternal education	No education	Ref		Ref	Ref
	Primary	0.60 (0.27 to 1.32)		0.65 (0.17 to 2.38)	0.97 (0.21 to 4.32)
	Secondary or higher	0.42 (0.19 to 0.91) *		0.22 (0.10 to 0.52) *	0.24 (0.07 to 0.81) *
Maternal working status	No	Ref		Ref	
	Yes	1.20 (0.59 to 2.45)		0.72 (0.29 to 1.75)	
Paternal education	No education	Ref		Ref	
	Primary	0.95 (0.35 to 2.58)		1.48 (0.31 to 7.14)	
	Secondary or higher	0.53 (0.26 to 1.07)		0.37 (0.16 to 0.89) *	
	No	Ref		Ref	

Paternal working status	Yes	7.17e-7 (0.00 to inf)		3.74e-7 (0.00 to inf)	
Family size	1 to 7 members	Ref		Ref	
	8 or more members	1.00 (0.53 to 1.87)		1.35 (0.62 to 2.90)	
Type of place of residence	Rural	Ref		Ref	
	Urban	0.77 (0.40 to 1.47)		0.28 (0.12 to 0.62) *	

∞ = The reference category for assessing the determinants of coexistence of underweight with stunting was underweight.

1 = Adjusted for socioeconomic status, and child age

2 = Adjusted for socioeconomic status, child age, and maternal education.

C: Assessing the determinants of coexistence of underweight with wasting and stunting both (underweight as reference category):

**Variables**	Categories	PDHS 2012-2013		PDHS 2017-2018	
		Unadjusted Odds (95% CI)	Adjusted odds [1] (95% CI)	Unadjusted Odds (95% CI)	Adjusted odds 2 (95% CI)
**Socioeconomic status**	Poorest	Ref	Ref	Ref	Ref
	Poorer	0.38 (0.14 to 1.05)	0.31 (0.10 to 0.97) *	1.06 (0.31 to 3.67)	0.76 (0.18 to 3.21)
	Middle	0.47 (0.15 to 1.46)	0.22 (0.06 to 0.82) *	0.33 (0.08 to 1.27)	0.59 (0.09 to 3.81)
	Richer	0.73 (0.23 to 2.29)	0.30 (0.06 to 1.33)	0.86 (0.18 to 4.01)	5.34 (0.45 to 62.81)
	Richest	0.17 (0.05 to 0.52) *	0.04 (0.008 to 0.21) *	0.05 (0.009 to 0.34) *	0.13 (0.01 to 1.36)
Sex	Male	Ref		Ref	
	Female	0.91 (0.45 to 1.81)		0.55 (0.23 to 1.31)	
Age	0-11 mo	Ref	Ref	Ref	Ref
	12-23 mo	6.63 (2.29 to 19.17) *	7.87 (2.49 to 24.87) *	14.31 (2.76 to 74.25) *	22.64 (2.33 to 219.63) *
	24-35 mo	6.57 (2.02 to 21.31) *	7.51 (2.14 to 26.28) *	4.77 (1.22 to 18.53) *	2.77 (0.60 to 12.76)
	36-47 mo	3.82 (1.33 to 10.94) *	5.73 (1.76 to 18.59) *	15.0 (2.9 to 77.59) *	27.44 (3.45 to 218.27) *
	48-59 mo	2.91 (0.95 to 8.89)	4.28 (1.19 to 15.32) *	5.18 (1.47 to 18.18) *	3.70 (0.82 to 16.58)
Maternal education	No education	Ref		Ref	Ref
	Primary	0.29 (0.11 to 0.77) *		0.29 (0.06 to 1.41)	0.33 (0.03 to 3.58)
	Secondary or higher	0.56 (0.24 to 1.30)		0.21 (0.07 to 0.54) *	0.11 (0.01 to 0.74) *

Maternal working status	No	Ref		Ref	
	Yes	0.98 (0.44 to 2.16)		0.62 (0.22 to 1.73)	
Paternal education	No education	Ref		Ref	
	Primary	0.92 (0.31 to 2.73)		1.66 (0.31 to 8.79)	
	Secondary or higher	0.51 (0.23 to 1.10)		0.42 (0.16 to 1.09)	
Paternal working status	No	Ref		Ref	
	Yes	5.05e-7 (0.00 to inf)		1.89e-7 (0.00 to inf)	
Family size	1 to 7 members	Ref		Ref	
	8 or more members	1.00 (0.50 to 2.00)		1.49 (0.63 to 3.52)	
Type of place of residence	Rural	Ref	Ref	Ref	
	Urban	1.24 (0.61 to 2.52)	4.05 (1.29 to 12.73) *	0.56 (0.23 to 1.37)	

∞ = The reference category for assessing the determinants of coexistence of underweight with wasting and stunting both was underweight.

1 = Adjusted for socioeconomic status, child age, and type of place of residence.

2 = Adjusted for socioeconomic status, child age, and maternal education.

D: Assessing the determinants of coexistence of Overweight/obesity with Stunting (stunting as reference category):

**Variables**	Categories	PDHS 2012-2013 Unadjusted Odds (95% CI)	PDHS 2012-2013 Adjusted odds [1] (95% CI)	PDHS 2017-2018 Unadjusted Odds (95% CI)	PDHS 2017-2018 Adjusted odds 2 (95% CI)
**Socioeconomic status**	Poorest	Ref	Ref	Ref	Ref
	Poorer	0.50 (0.31 to 0.81) *	0.39 (0.23 to 0.66) *	1.53 (0.53 to 4.42)	1.45 (0.49 to 4.33)
	Middle	0.52 (0.31 to 0.87) *	0.43 (0.25 to 0.76) *	1.38 (0.45 to 4.23)	1.54 (0.48 to 4.86)
	Richer	0.40 (0.23 to 0.69) *	0.34 (0.19 to 0.61) *	2.27 (0.76 to 6.79)	2.14 (0.69 to 6.65)
	Richest	1.26 (0.75 to 2.12)	1.05 (0.60 to 1.83)	6.66 (2.48 to 17.90) *	5.80 (2.07 to 16.27) *
Sex	Male	Ref		Ref	
	Female	1.05 (0.75 to 1.48) *		1.32 (0.72 to 2.43)	
Age	0-11 mo	Ref	Ref	Ref	Ref
	12-23 mo	0.13 (0.06 to 0.28) *	0.13 (0.06 to 0.29) *	0.08 (0.02 to 0.26) *	0.09 (0.03 to 0.30) *

	24-35 mo	0.13 (0.07 to 0.26) *	0.13 (0.06 to 0.26) *	0.22 (0.09 to 0.52) *	0.23 (0.09 to 0.56) *
	36-47 mo	0.24 (0.13 to 0.45) *	0.23 (0.12 to 0.45) *	0.04 (0.01 to 0.16) *	0.05 (0.01 to 0.19) *
	48-59 mo	0.28 (0.15 to 0.52) *	0.29 (0.15 to 0.55) *	0.12 (0.04 to 0.32) *	0.14 (0.05 to 0.40) *
Maternal education	No education	Ref		Ref	
	Primary	0.94 (0.58 to 1.53)		0.91 (0.31 to 2.75)	
	Secondary or higher	1.09 (0.73 to 1.63)		2.51 (1.32 to 4.75) *	
Maternal working status	No	Ref	Ref	Ref	
	Yes	0.56 (0.35 to 0.88) *	0.51 (0.31 to 0.84) *	0.15 (0.02 to 1.11)	
Paternal education	No education	Ref		Ref	
	Primary	0.97 (0.56 to 1.69)		0.59 (0.15 to 2.25)	
	Secondary or higher	1.19 (0.81 to 1.73)		2.76 (1.29 to 5.91) *	
Paternal working status	No	Ref	Ref	Ref	
	Yes	0.27 (0.11 to 0.66) *	0.31 (0.11 to 0.82) *	3.80e+6 (0.00 to inf)	
Family size	1 to 7 members	Ref		Ref	
	8 or more members	1.31 (0.92 to 1.87)		0.85 (0.46 to 1.56)	
Type of place of residence	Rural	Ref		Ref	
	Urban	1.29 (0.92 to 1.82)		2.19 (1.18 to 4.06) *	

∞ = The reference category for assessing the determinants of coexistence of stunting with overweight/obesity was stunting.

1 = Adjusted for socioeconomic status, child age, maternal work status, and paternal work status.

2 = Adjusted for socioeconomic status, and child age

Supplementary file S4

Table S4: Prevalence of various types of malnutrition in children of in Azad Jammu Kashmir (AJK) and Federally Administered Tribal Areas (FATA) region using PDHS-2017-2018

Malnutrition type	AJK	FATA
**Overall malnutrition**	30.8% (26.3 to 35.7%)	55.5% (49.9 to 61%)
**Standalone forms of malnutrition**	20.5% (16.6 to 24.8%)	28.5% (23.6 to 33.7)
**Coexisting forms of malnutrition**	10.3% (7.6 to 13.8%)	27% (22.5 to 32.1%)
**Coexistence of undernutrition**	9.9% (7.1 to 13.2%)	23.0% (18.5 to 27.9%)
**Coexistence of stunting with overweight/obesity**	0.5% (0.06 to 1.8%)	4.0% (2.1 to 6.7%)
**Coexistence of underweight with stunting**	7.4% (5.2 to 10.6%)	17.8% (13.7 to 22.3%)
**Coexistence of underweight with wasting**	1.5% (0.5 to 3.2%)	2.5% (1.1 to 4.7%)
**Coexistence of underweight with wasting and stunting**	0.8% (0.2 to 2.2%)	2.8% (1.2 to 5.1%)

Where, AJK = Azad Jammu Kashmir, FATA = Federally Administered Tribal Areas.
